# Infection with the Lyme disease pathogen suppresses innate immunity in mice with diet‐induced obesity

**DOI:** 10.1111/cmi.12689

**Published:** 2016-11-25

**Authors:** Nataliya Zlotnikov, Ashkan Javid, Mijhgan Ahmed, Azad Eshghi, Tian Tian Tang, Anoop Arya, Anil Bansal, Fatima Matar, Maitry Parikh, Rhodaba Ebady, Adeline Koh, Nupur Gupta, Peng Song, Yang Zhang, Susan Newbigging, Gary P. Wormser, Ira Schwartz, Robert Inman, Michael Glogauer, Tara J. Moriarty

**Affiliations:** ^1^ Matrix Dynamics Group, Faculty of DentistryUniversity of TorontoTorontoOntarioCanada; ^2^ Mount Sinai Hospital Research Institute/Toronto Centre for PhenogenomicsTorontoOntarioCanada; ^3^ Division of Infectious DiseasesNew York Medical CollegeNew YorkUSA; ^4^ Department of Microbiology and ImmunologyNew York Medical CollegeNew YorkUSA; ^5^ Department of Immunology, Faculty of MedicineUniversity of Toronto, Toronto Hospital—Western DivisionTorontoOntarioCanada; ^6^ Department of Laboratory Medicine and Pathobiology, Faculty of MedicineUniversity of TorontoTorontoOntarioCanada

**Keywords:** Borrelia burgdorferi, diseases, host obesity, immunology, infection, Lyme disease

## Abstract

Obesity is a major global public health concern. Immune responses implicated in obesity also control certain infections. We investigated the effects of high‐fat diet‐induced obesity (DIO) on infection with the Lyme disease bacterium *Borrelia burgdorferi* in mice. DIO was associated with systemic suppression of neutrophil‐ and macrophage‐based innate immune responses. These included bacterial uptake and cytokine production, and systemic, progressive impairment of bacterial clearance, and increased carditis severity. *B. burgdorferi*‐infected mice fed normal diet also gained weight at the same rate as uninfected mice fed high‐fat diet, toll‐like receptor 4 deficiency rescued bacterial clearance defects, which greater in female than male mice, and killing of an unrelated bacterium (Escherichia coli) by bone marrow‐derived macrophages from obese, *B. burgdorferi*‐infected mice was also affected. Importantly, innate immune suppression increased with infection duration and depended on cooperative and synergistic interactions between DIO and *B. burgdorferi* infection. Thus, obesity and *B. burgdorferi* infection cooperatively and progressively suppressed innate immunity in mice.

## 1 INTRODUCTION

Obesity is a major global public health concern (Finucane et al*.*, 2011). Innate immune responses that regulate the development of obesity also control certain infections (Jin, Henao‐Mejia, & Flavell, 2013; McNelis & Olefsky, 2014), and obesity is associated with increased risk, morbidity, and fatality due to some infections in humans (Dhurandhar, Bailey, & Thomas, 2015). In mouse models of human obesity (high‐fat diet [HFD]‐induced obesity [DIO]), obesity impairs immune responses to Staphylococcus aureus and *Porphyromonas gingivalis* (Amar, Zhou, Shaik‐Dasthagirisaheb, & Leeman, 2007; Yano et al*.*, 2012; Farnsworth et al*.*, 2015). However, the impact of obesity on infection and effects of infection on obesity are still not well understood.

Innate immune sensing of metabolic conditions, tissue damage, and microbes is mediated by many of the same pattern recognition receptors and associated signaling and cell activation pathways, particularly in macrophages (Jin et al*.*, 2013; McNelis & Olefsky, 2014). Not surprisingly, obesity and associated metabolic syndrome are dependent on and characterized by dysregulation of many features of innate immunity, including changes in the function and relative abundance of different types of innate immune cells (Jin et al*.*, 2013; McNelis & Olefsky, 2014). Conversely, altered composition of the microbiota and increased microbial penetration of mucosal barriers under high‐fat dietary conditions is thought to contribute to increased basal stimulation and disruption of innate immune receptor signaling (Rosenbaum, Knight, & Leibel, 2015).

Obesity is associated with hypertension, hyperglycemia, hyperlipidemia, and hypercholesterolemia. We and others have recently found that obesity‐independent hyperglycemia and obesity‐independent hypercholesteremia in mice are associated with disrupted innate and adaptive immune responses to the Lyme disease pathogen *Borrelia burgdorferi* and impaired control of bacterial burden and/or *B. burgdorferi* DNA clearance from tissues (Toledo, Monzón, Coleman, Garcia‐Monco, & Benach, 2015; Javid et al*.*, 2016). Therefore, metabolic conditions associated with obesity can affect *B. burgdorferi* infection in mouse models. Infection with *B. burgdorferi* has been increasing in incidence in parallel with obesity in much of the industrialized world (Finucane et al*.*, 2011; Mead, 2015). Approximately 300,000 *B. burgdorferi* infections occur annually in the United States (Mead, 2015), but the potential effects of obesity itself on *B. burgdorferi* infection and Lyme disease outcomes have not been examined. The purpose of the present study was to evaluate the potential interplay of obesity and infection in mouse models of *B. burgdorferi* infection.

We investigated the effects of HFD feeding (diet‐induced obesity [DIO]) on *B. burgdorferi* infection, infection‐dependent pathologies and innate immune responses in mice. DIO in mice is the animal model most commonly used to investigate mechanisms underlying human obesity and associated metabolic syndrome (Wang & Liao, 2012). Our results indicated that unlike for obesity‐independent hyperglycemia, DIO was associated with more severe infection‐dependent pathology and widespread inhibition of innate immune responses to *B. burgdorferi* infection and that innate immune suppression in DIO was dependent on *B. burgdorferi* infection itself.

## 2 RESULTS

To determine if DIO affected *B. burgdorferi* infectivity, infection outcomes and innate immune responses in mice, we conducted most of our studies in male C3H/HeN mice infected with 1 × 10^4^ of B31 5A4‐derived *B. burgdorferi*. However, mouse strains differ in the severity of pathology induced by *B. burgdorferi* infection, and *B. burgdorferi* strains differ in their pathogenicity and tissue tropism patterns. Therefore, we also examined a subset of experimental parameters (bacterial DNA copy number and carditis) in several mouse strains and for four distinct *B. burgdorferi* strains, as well as in both male and female mice, as described below. This approach permitted us to determine if DIO had effects on *B. burgdorferi* infection that were observed across multiple host and bacterial genetic backgrounds. It also permitted identification of *B. burgdorferi* and mouse strains and tissues that were most affected by DIO. Figure S1a provides details of all mouse and bacterial strains used in these studies and a flowchart summarizing dietary preconditioning and infection timelines of experiments. It is important to note that due to the duration of dietary preconditioning (8–12 weeks), all mice were mature adults at the time of infection (13 weeks for C57 and 16–18 weeks for C3H), and were thus considerably older than the juvenile animals (3–5 weeks) typically used in *B. burgdorferi* infectivity studies, which are more susceptible to the development of Lyme disease pathology than older animals (Barthold, Cadavid, & Philipp, 2010).

We investigated the effects of DIO on *B. burgdorferi* infection in the most common mouse models of DIO (male C57BL/6) (Wang & Liao, 2012) and Lyme disease (male and female C3H) (Barthold et al*.*, 2010). C57BL/6 and C3H mice are respectively resistant and susceptible to the development of *B. burgdorferi*‐induced carditis and arthritis (Barthold et al*.*, 2010). Experiments were also performed comparing wild‐type C3H/HeN and toll‐like receptor 4 (TLR4)‐deficient C3H/HeJ male mice to determine whether TLR4 status affected *B. burgdorferi* infection in DIO. TLR4 is not required for immune responses to *B. burgdorferi* infection *in vivo*, but contributes to *B. burgdorferi*‐elicited cytokine secretion in macrophages, promotes the development of insulin‐resistant hyperglycemia in response to HFD, and is a key metabolic sensor of lipids (Glickstein & Coburn, 2006; Weis & Bockenstedt, 2010; Jin et al*.*, 2013; Cervantes et al*.*, 2014; Petnicki‐Ocwieja & Kern, 2014). In addition, we compared effects of DIO in male and female C3H/HeN mice, because sexual dimorphism in innate and adaptive immunity results in sexually dimorphic outcomes in certain infections, as well as in DIO (Markle & Fish, 2014; Singer et al*.*, 2015). Finally, DIO effects were also examined in male HeN mice infected with four *B. burgdorferi ospC* strain types associated with disseminated infection in U.S. clinical populations, GCB726 (B31 5A4 NP1, *ospC*‐type A), BL214 (*ospC*‐type B), N40 (*ospC*‐type E), and 297 (*ospC*‐type K) (Petzke & Schwartz, 2015).

### 2.1 Weight gain in mice fed HFD and following B. burgdorferi infection in ND groups

**Figure 1:**
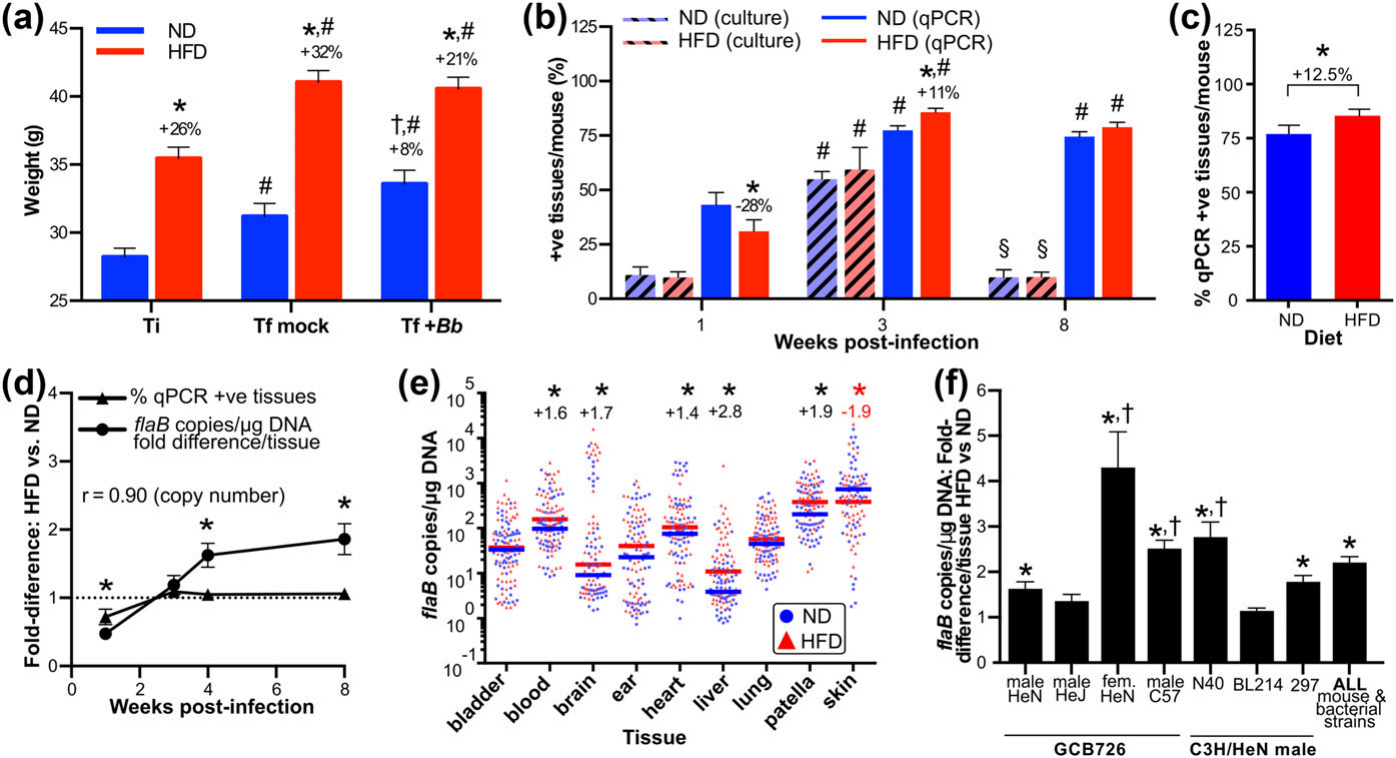
Progressive, systemic, and toll‐like receptor (TLR)4‐dependent impairment of *Borrelia* *burgdorferi* clearance in diet‐induced obesity (DIO). (a) Body weight at time of infection (*T*_*i*_) and final sacrifice (*T*_*f*_) for mice preconditioned with normal (ND) or high‐fat diet (HFD) and infected for 4 weeks with 1 × 10^4^ *B. burgdorferi* (+*Bb*) or vehicle (mock: bacterial cultivation medium alone). Mean ± 95% confidence intervals (CIs) are shown for all mouse sexes and strains and bacterial strains analyzed in this study. At *T*_*i*_, all HFD mice were ≥25% heavier than age‐, sex‐, and strain‐matched ND controls and were obese. *N* ≥ 30 (*T*_*i*_) and 98 mice (in each of *T*_*f*_ mock and *T*_*f*_ + *Bb* groups) per diet group. * indicates *p* < 0.05 HFD versus ND within time point (% difference shown above HFD columns). † indicates *p* < 0.05 *T*_*f*_ + *Bb* versus *T*_*f*_ mock for ND (% difference shown above *T*_*f*_ + *Bb* column). # indicates *p* < 0.05 *T*_*f*_ versus *T*_*i*_ within diet. Weight and blood glucose values for individual experiments are provided in Figure S1c–f. (b) Mean ± standard error of the mean (*SEM*) percentage of tissues from male C3H/HeN mice infected with 1 × 10^4^ GCB726 from which *B. burgdorferi* could be cultivated (culture) or that were positive for *B. burgdorferi flaB* DNA (quantitative polymerase chain reaction [qPCR]) 1, 3, and 8 weeks after infection. *N* = 10–11 mice per diet group (Weeks 1 and 8) and 5 mice per diet group (Week 3). Values above columns indicate percentage change in qPCR‐positive tissues in mice for HFD versus ND at the same time point. * indicates *p* < 0.05 HFD versus ND within time point. # indicates *p* < 0.05 3 and 8 weeks versus 1 week within diet group. § indicates 8 versus 3 weeks within diet group. (c) Mean (± 95% CI) percentage of qPCR‐positive tissues per mouse for all mouse strains and sexes and bacterial strains combined. * indicates *p* < 0.05 (two‐tailed *t*‐test). Values for individual experiments are provided in Figure S2b. (d) Mean ± *SEM* fold‐differences (HFD vs. ND) in percentage of qPCR‐positive tissues and median *flaB* copy number/tissue 1, 3, 4, and 8 weeks after infection of male C3H/HeN mice with 1 × 10^4^ GCB726. Average fold differences in median *flaB* copy number/tissue were calculated by normalizing DNA concentration‐adjusted values for each tissue from each mouse to the median *flaB* copy number for the same tissue from ND mice. Then, for each HFD mouse, median fold differences obtained for each tissue were averaged to obtain the mean fold difference/tissue across all tissues. Shown in panel D are the means of mean fold difference/tissue for all mice within the HFD group. *r*: Pearson correlation coefficient: copy number fold difference versus time. *N* = 5–11 mice/diet group and time point. * indicates *p* < 0.05 copy number versus % qPCR‐positive fold differences. Raw values are provided in Figure S2c–d. (e) Median *flaB* copy number in individual tissues for all mouse strains and sexes and bacterial strains combined. Each data point corresponds to the value for one tissue from one mouse 4 weeks post‐infection. Values above datasets for each tissue indicate significant fold‐differences in HFD versus ND group (* *p* < 0.05). Raw values for individual experiments are provided in Figure S2e–k. Each diet group for each experimental group contained 9–11 mice. Statistical comparison between diet groups for this meta‐analysis was performed by two‐way ANOVA, and each individual experimental group was equally weighted, to control for differences in mouse numbers in individual groups. (f) Mean ± *SEM* fold‐differences (HFD vs. ND) in median bacterial burden/tissue for individual mouse strains and sexes and bacterial strains. HeJ mice are TLR4‐deficient. *N* ≥ 9 mice/diet group for individual experiments. *N* = 9–11 mice/diet group and experimental condition. * indicates *p* < 0.05 HFD versus ND. † indicates *p* < 0.05 versus male HeN infected with GCB726

At time of infection (*T*_*i*_) all mice fed HFD weighed 26% more on average than mice fed control normal diet (ND) and were thus obese (Figure 1a) (Wang & Liao, 2012). *Ad libitum* (non‐fasting) blood glucose levels in mice fed HFD were on average 10–20% greater than in ND counterparts (corresponding to mild hyperglycemia) and were not markedly affected by *B. burgdorferi* infection (Figure S1b). These increases in blood glucose were approximately 15–30 times smaller than the increases observed in obesity‐independent hyperglycemia models, where blood glucose typically increases by more than threefold compared to normoglycemic mice (Javid et al*.*, 2016). Surprisingly, infected mice fed ND weighed 8% more (*p* < 0.05) on average than mock‐infected controls at time of sacrifice (Figure 1a). A similar weight increase of ~10% was observed in ND infected mice from individual experimental groups, although this weight gain was not significant in all experimental groups (Figure S1c,e). This corresponded to an average weight gain of 2% per week, which was comparable to the rate of weight gain in C3H animals fed HFD during preconditioning (2.2% per week). Thus, *B. burgdorferi* infection promoted weight gain as effectively as HFD.

### 2.2 Progressive, systemic, TLR4‐dependent impairment of *B. burgdorferi* clearance in DIO

To characterize the effects of DIO on *B. burgdorferi* infection, we determined if bacterial infectivity, percentage of tissues colonized/mouse (dissemination), persistence of infection, and bacterial DNA load/tissue differed in ND and HFD mice. For these experiments, nine tissues were examined for each mouse (bladder, blood, brain, ear, heart, knee joint (patella), liver, lung, and skin). Infectious doses (ID_50_) were similar in mice fed ND and HFD (*p* > 0.05; Figure  S2a), and the percentage of tissues/mouse from which *B. burgdorferi* could be cultivated did not differ between diet groups over the course of infection (*p* > 0.05; Figure 1b). Thus, DIO did not affect *B. burgdorferi* infectivity or persistence of cultivatable bacteria.

We also evaluated dissemination and persistence by measuring the percentage of tissues that were positive for *B. burgdorferi flaB* DNA by quantitative polymerase chain reaction (qPCR) (Figure 1b–c). Although fewer tissues were qPCR positive in DIO during early infection (1 week), by 3 weeks post‐infection (acute, post‐dissemination phase), more tissues were qPCR‐positive in HFD than in ND animals (Figure 1b). Similar increases in the percentage of qPCR‐positive tissues in DIO were observed at 4 weeks post‐infection across all mouse strains and sexes and bacterial strains examined in our studies (Figure 1c). By late infection (8 weeks), although *B. burgdorferi* could be cultivated from only a small number of tissues, the percentage of tissues that were qPCR‐positive remained high (Figure 1b), possibly reflecting incomplete clearance of bacterial debris or the presence of uncultivatable bacteria (Bockenstedt, Gonzalez, Haberman, & Belperron, 2012; Hodzic, Imai, Feng, & Barthold, 2014). The percentage of qPCR‐positive tissues did not differ in ND and HFD groups at 8 weeks post‐infection (*p* > 0.05; Figure 1b). Together, these results indicated that DIO did not affect *B. burgdorferi* infectivity or persistence of cultivatable bacteria, but was associated with small but significant increases in numbers of qPCR‐positive tissues at the acute (3–4 weeks) phase of infection.

Although the percentage of *B. burgdorferi*‐positive tissues did not increase after 3–4 weeks, average *flaB* copy number/tissue increased in HFD compared to ND groups as infection progressed, with the greatest fold difference in copy number/tissue observed at 8 weeks (Figure 1d). Because increased copy number at 8 weeks was not accompanied by increased frequency of qPCR‐positive or culture‐positive tissues in DIO (Figure 1b,d), it was likely that elevated load reflected impaired clearance of non‐cultivatable bacteria and/or bacterial debris from tissues and not greater bacterial proliferation or dissemination.

Consistent with known differences in tissue tropism among *B. burgdorferi* strains, the distribution of *flaB* copy number among the nine tissues examined varied for different bacterial strains and mouse strains and sexes, in both ND and HFD groups (Figure S2e–k). To identify the tissues where *flaB* copy number was most affected by DIO across all experimental groups, independent of differences in tissue tropism among groups—that is, to identify where the effects of DIO were strongest across all experiments, we compared copy number in all tissues from all experimental groups (Figure 1e). The primary data for all individual experiments are presented in Figure S2e–k. Across all mouse strains and sexes and bacterial strains, DIO was associated with significant increases in bacterial DNA burden in blood, brain, heart, liver, and knee joint (patella) compared to ND controls (Figures 1e and S2e–k). The only tissue where bacterial burden was globally reduced across all HFD groups was skin (Figure 1e). DIO induces macrophage‐based hyperinflammatory responses in the skin of mice (Zhang et al*.*, 2015) that possibly accounted for enhanced *B. burgdorferi* clearance from the skin in our experiments. Thus, DIO was associated with progressive, systemic impairment of *B. burgdorferi* clearance in multiple tissues.

To compare the effects of DIO on bacterial DNA copy number for different mouse strains, sexes, and *B. burgdorferi* strain, we calculated the median HFD:ND copy number fold difference in each tissue, then compared the average of fold differences in all tissues (fold difference/tissue) across all experimental groups (Figure 1f). Overall, DIO was associated with a significant 2.2‐fold increase in bacterial DNA copy number per tissue across all mouse strains, sexes, and *B. burgdorferi* strains (Figure 1f). This effect was most pronounced for female mice (Figure 1f), even though colonization frequency and bacterial DNA load across all tissues did not differ for male and female mice under normal dietary conditions (*p* > 0.05; Figure S2b,e,j). Consistent with previous reports (Weis & Bockenstedt, 2010), TLR4 deficiency in HeJ mice did not affect global colonization frequency or bacterial DNA load across all tissues under ND conditions (*p* > 0.05; Figure S2e,k). However, TLR4 deficiency protected against DIO‐dependent increases in bacterial copy number (Figure 1f: compare male HeN and HeJ groups infected with GCB726), indicating that TLR4 function contributed to impaired bacterial clearance in DIO. Collectively, these data showed that DIO was associated with progressive, systemic, and TLR4‐dependent impairment of *B. burgdorferi* clearance and that this effect was sexually dimorphic.

### 2.3 Increased Lyme carditis severity in DIO

**Figure 2:**
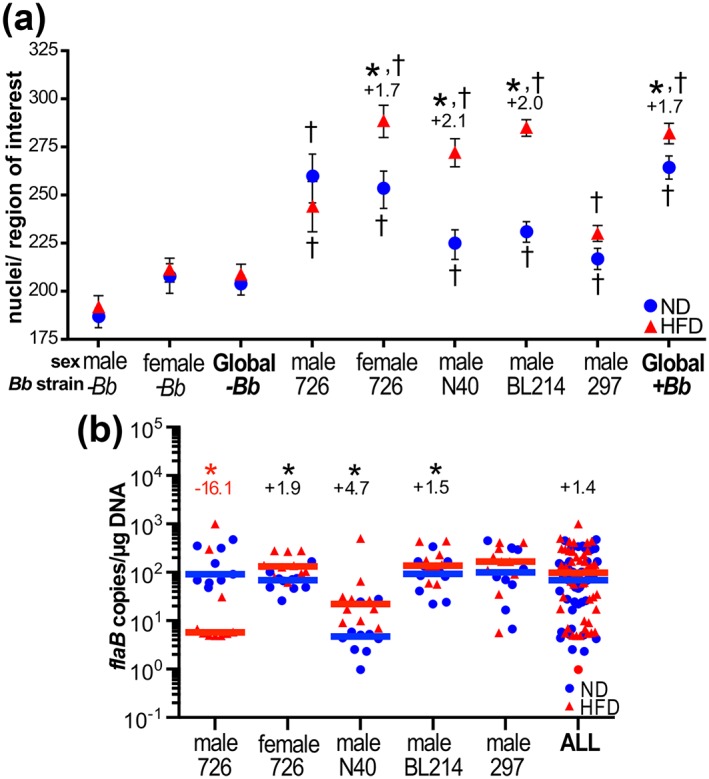
Increased Lyme carditis severity in diet‐induced obesity (DIO). (a) Mean ± standard error of the mean (*SEM*) carditis (inflammation) scores, calculated as described in experimental procedures by measuring mean numbers of nuclei/region of interest in heart sections from mice infected with indicated bacterial strains, and age‐, sex‐, and strain‐matched mock‐infected mice (−*Bb*). Global values (−*Bb* and +*Bb*) are means for all sexes and bacterial strains combined. Representative histology images are provided in Figure S3. (b) Median *Borrelia burgdorferi* DNA copy number in hearts for indicated mouse strains and sexes and bacterial strains, and all groups (ALL). Each data point corresponds to the copy number values for one mouse. For a and b, values above datasets indicate significant fold‐differences for high‐fat diet (HFD) versus normal diet (ND) groups. *N* ≥ 9 mice/diet group for individual experiments. * indicates *p* < 0.05 HFD versus ND. † indicates *p* < 0.05 versus age‐, sex‐, and diet‐matched mock‐infected control group († symbols placed above and below mean values for each infected diet group)

To determine if DIO affected the pathological outcomes of *B. burgdorferi* infection, we evaluated inflammation in tibiotarsal joint, heart, brain, and liver. No overt, infection‐dependent differences in inflammation were observed in joints, brain, or liver of either ND or HFD mice under multiple experimental conditions (mouse strains, sexes, and bacterial strains), as determined by a murine veterinary pathologist (data not shown). *B. burgdorferi*‐dependent arthritis was presumably not detected in either diet group because animals were considerably older than the optimal age for induction of this pathology in mice (3 weeks) (Barthold et al*.*, 2010). However, inflammation was significantly elevated in the hearts of mice from experimental groups where bacterial DNA burden was increased in DIO (Figure 2).

Prolonged hyperglycemia, which frequently accompanies DIO, can result in reduced cellular density in cardiac tissues as a result of excess extracellular matrix accumulation (Bugger & Abel, 2009). Inflammation in cardiac tissues from hyperglycemic mice must therefore be measured using approaches that take into account potential changes in cell density (cellularity). To measure carditis, we adapted a method for quantifying multifocal cardiac inflammation that entails counting nuclei/field of view in multiple sections (Diebold et al*.*, 1995). Using this method, we found that hearts of mock‐ and *B. burgdorferi*‐infected HFD mice were not hypocellular (Figure 2a), although unstained globular structures suggestive of fat deposits and/or adipocytes were sometimes observed (Figure S3a). *B. burgdorferi*‐infected animals in all experimental and diet groups exhibited significant cardiac hypercellularity compared to mock‐infected controls, indicating the presence of infection‐induced infiltration and proliferation of inflammatory cells (Figure 2a). Comparison of nuclei counts with *flaB* copy number (Figure 2b) revealed a striking correspondence between bacterial DNA burden and hypercellularity in hearts of ND and HFD mice. Hypercellularity was significantly elevated in all HFD infected groups where *flaB* copy number was greater than in ND infected controls—that is, in GCB726‐infected female mice and N40‐ and BL214‐infected male mice—and was greater in GCB726‐infected ND male mice compared to HFD counterparts, where cardiac burden was higher under ND than HFD conditions (Figure 2a,b). Thus, infection‐dependent carditis was more severe in the hearts of mice where DIO impaired bacterial clearance.

### 2.4 Attenuated and delayed systemic neutrophil responses to B. burgdorferi infection in DIO

**Figure 3:**
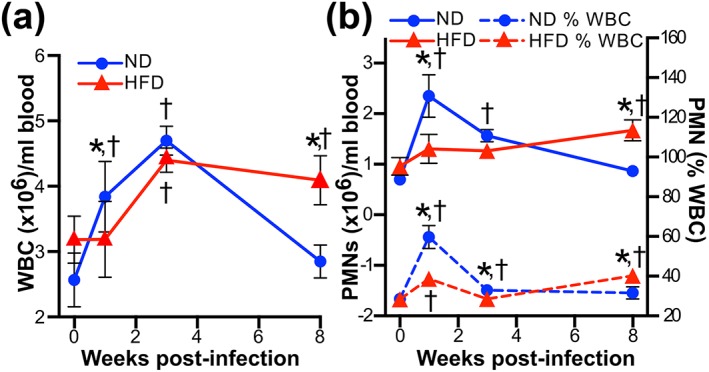
Attenuated and delayed systemic neutrophil responses to *Borrelia burgdorferi* infection in diet‐induced obesity (DIO). Mean ± standard error of the mean (*SEM*) cell count/ml blood for white blood cells (a: WBC) and neutrophils (b: PMN) 0, 1, 3, and 8 weeks after infection (0 weeks: uninfected baseline). Experiments were performed with male C3H/HeN mice infected with 1 × 10^4^ GCB726. The percentage of total WBCs that were PMNs is indicated by dashed lines plotted on the right *y*‐axis. Blood counts for other WBC types are provided in Figure S3b–e. *N* = 10–11 mice per diet group (Weeks 0, 1, and 8) and 5 mice per diet group (week 3). * indicates *p* < 0.05 normal diet (ND) versus high‐fat diet (HFD). † indicates infected versus uninfected (0 weeks) within diet group

Because DIO affected *B. burgdorferi* clearance in multiple tissues and DIO alters innate immune responses (Jin et al*.*, 2013), we next asked whether systemic responses were altered in HFD animals, by measuring absolute concentrations of total white blood cells (WBCs), neutrophils (PMNs), and other WBC types in whole blood. We recently found that obesity‐independent hyperglycemia impairs innate immune responses to male C3H/HeN mice infected with 1 × 10^4^ of *B. burgdorferi* strain GCB726 (Javid et al*.*, 2016). Therefore, experiments investigating the effects of DIO on innate immune responses to *B. burgdorferi* infection were also performed with male C3H/HeN mice infected with 1 × 10^4^ GCB726, to facilitate comparison of the effects of obesity and hyperglycemia on innate immune responses. In ND mice, WBC numbers increased significantly at 1 week post‐infection compared to baseline (0 weeks), peaked at the acute phase of infection (3 weeks), and subsequently declined, consistent with a systemic response to *B. burgdorferi* infection (Figure 3a). By contrast, in DIO, systemic WBC responses to infection were delayed and remained significantly elevated late in infection compared to baseline and ND infected mice. Therefore, both activation and resolution of systemic inflammation in response to *B. burgdorferi* were delayed. The types of circulating WBCs associated with systemic responses to *B. burgdorferi* infection in DIO also differed markedly. The most prominently affected cell type was PMNs, which were especially abundant at 1 week post‐inoculation in ND animals, but declined to baseline levels by 8 weeks post‐infection (Figure 3b). In obese mice, PMN counts were not significantly elevated above baseline until 8 weeks post‐infection and even at this time point did not reach the same numbers as observed during the peak systemic PMN response in ND (Figure 3b). Instead, systemic cellular responses to *B. burgdorferi* infection in DIO were skewed toward eosinophilia and monocytosis (Figure S3b–c). Thus, DIO was associated with delayed and attenuated increases in systemic WBC and neutrophil numbers early in *B. burgdorferi* infection and blunted neutrophilia late in infection, accompanied by eosinophilia and monocytosis.

### 2.5 Progressive, global suppression of systemic cytokine responses to B. burgdorferi infection in DIO

**Figure 4:**
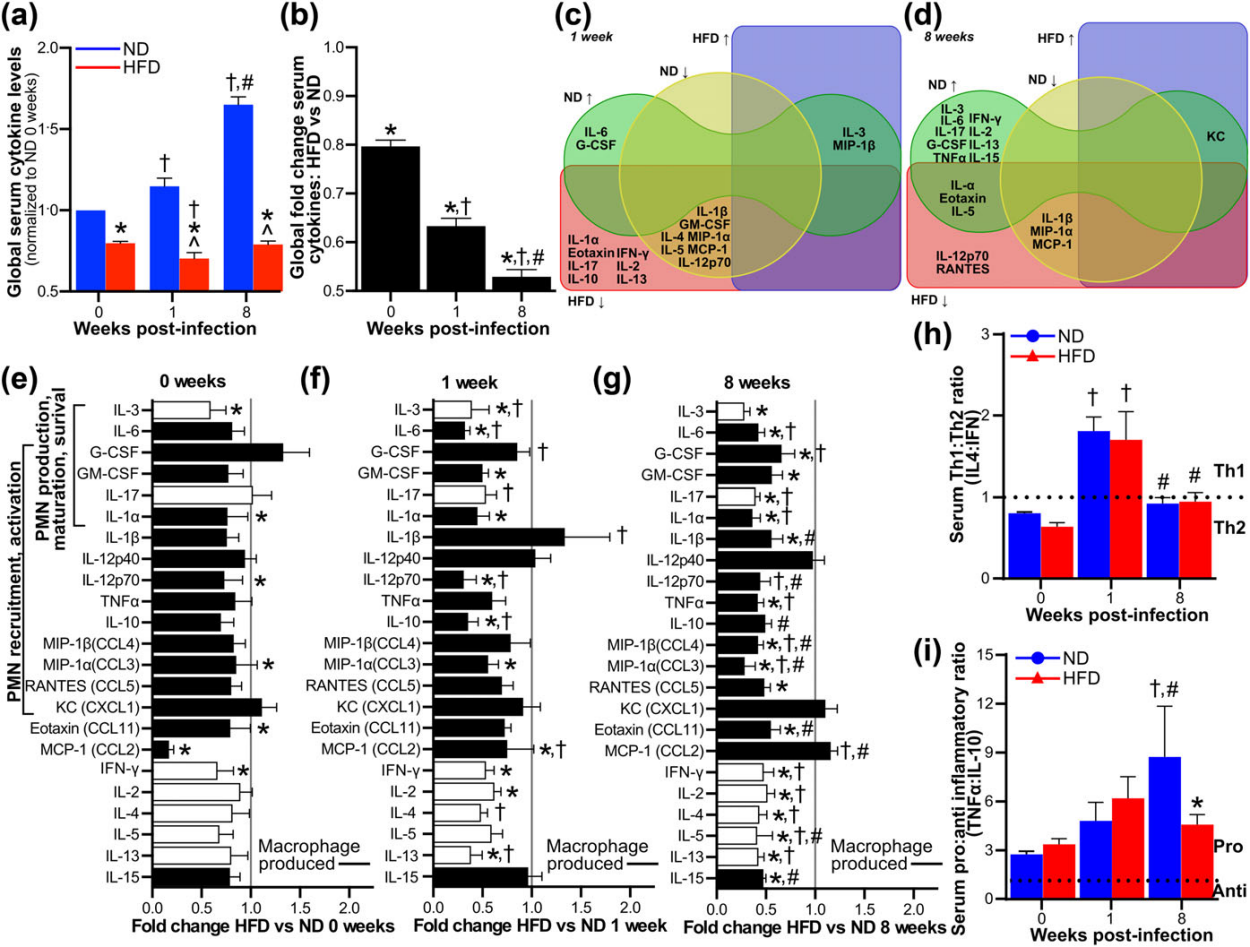
Progressive, global suppression of systemic cytokine responses to *Borrelia burgdorferi* infection in diet‐induced obesity (DIO). Serum levels of 23 cytokines 0, 1, and 8 weeks after infection (0 weeks: uninfected baseline) of male C3H/HeN mice with 1 × 10^4^ GCB726. (a–b) Global fold difference/cytokine for all cytokines, compared to normal diet (ND) uninfected baseline (a) and for high‐fat diet (HFD) versus ND (b). (c–d) Venn diagrams illustrating significantly (*p* < 0.05) upregulated (↑) or downregulated (↓) cytokines compared to ND uninfected baseline, at 1 (c) and 8 (d) weeks post‐infection. (e–g) HFD:ND fold‐differences for individual cytokines at 0 (e), 1 (f), and 8 (g) weeks. Cytokines involved in neutrophil production, maturation, survival, recruitment, and activation are indicated. Black bars indicate macrophage‐produced cytokines. (h) Th1:Th2 (interleukin [IL]‐4:interferon [IFN]‐γ) and (i) pro‐inflammatory: anti‐inflammatory (tumor necrosis factor [TNF]α:IL‐10) cytokine ratios. Raw data for all panels in this figure and names of cytokines indicated by abbreviations are provided in Figure S4. *N* = 10–11 mice per diet group and time point. Data summaries: mean ± standard error of the mean (*SEM*). * indicates *p* < 0.05 HFD versus ND. † indicates *p* < 0.05 infected versus uninfected (0 weeks) within diet group. # indicates *p* < 0.05 8 versus 1 week within diet group. ^ indicates *p* < 0.05 versus ND uninfected (0 weeks)

To determine if DIO also disrupted systemic cytokine responses to *B. burgdorferi* infection, we compared levels of 23 cytokines and chemokines in sera from early and late infection (1 and 8 weeks) to baseline values for mock‐infected ND controls at Day 0. Global levels of the 23 measured cytokines increased progressively over the course of infection in ND but not HFD animals (Figure 4a–b), indicating that normal systemic cytokine responses to infection were blunted in DIO. As reported previously (Isogai et al*.*, 1996; Benhnia et al*.*, 2005), interleukin (IL)‐1α, IL‐6, tumor necrosis factor (TNF)‐α, and interferon (IFN)‐γ levels were significantly upregulated upon infection of ND mice, as were many others (Figure 4c–d). Unexpectedly, multiple cytokines were also downregulated in this group compared to baseline, including IL‐1β (Figure 4c–d). By contrast, most cytokines upregulated in infected ND mice were not significantly upregulated in HFD animals following infection (Figure 4c–d), and the abundance of most cytokines declined significantly and progressively over the course of infection in HFD compared to normal groups (Figure 4e–g). Th1:Th2 cytokine ratios, estimated by comparing IL‐4:IFN‐γ abundance in the serum of each mouse, were similar in ND and HFD groups, suggesting that skewing of Th1/Th2 responses was not the major determinant of attenuated cytokine production in infected HFD mice (Figure 4h). However, TNF‐α:IL‐10 ratios in individual serum samples were significantly lower in HFD compared to ND mice at 8 weeks post‐infection (Figure 4i), primarily due to reduced levels of TNF‐α, and not increased IL‐10 abundance (Figures 4g and S4). Thus, DIO was associated with widespread attenuation and/or absence of normal systemic cytokine responses to *B. burgdorferi* infection.

We also noted that many of the cytokines that were less abundant in sera of infected HFD mice are macrophage‐produced (Figure 4f–g) (McNelis & Olefsky, 2014; Varol, Mildner, & Jung, 2015), suggesting that attenuated systemic cytokine production in these animals was possibly due, at least in part, to macrophage dysfunction. To test this hypothesis, we measured cytokines produced by peritoneally recruited macrophages (PMs) from infected mice and mock controls (Figure S5). The majority of cytokines were significantly reduced for PMs from infected HFD mice (Figure S5a), and many individual cytokines that were less abundant in sera of infected HFD animals (Figure 4f–g) were also produced less abundantly by PMs from these mice (Figure S5). This suggested that macrophage dysfunction was likely central to attenuated systemic cytokine responses to infection in DIO.

### 2.6 B. burgdorferi infection‐dependent suppression of neutrophil and macrophage bacterial uptake in DIO

**Figure 5:**
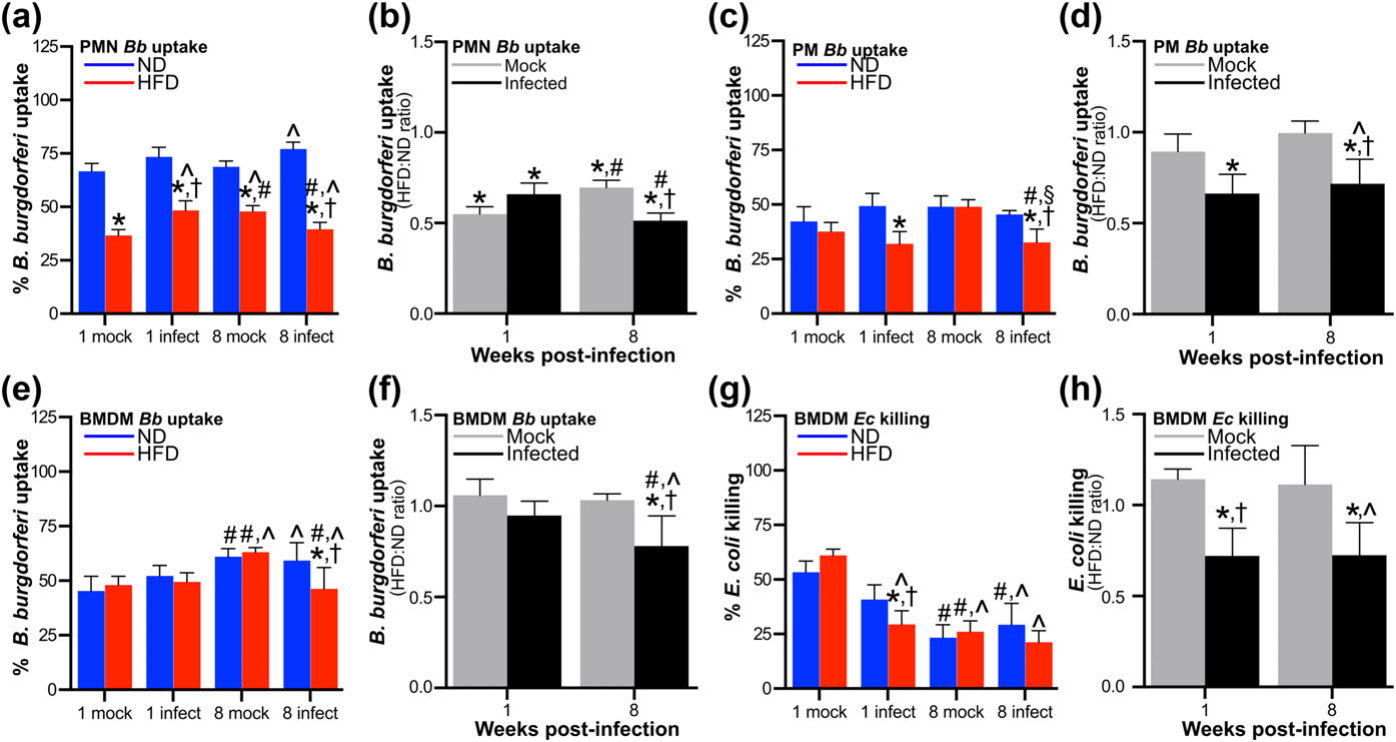
*Borrelia burgdorferi* infection‐dependent suppression of neutrophil and macrophage bacterial uptake in diet‐induced obesity (DIO). (a–f) *Ex vivo* uptake of *B. burgdorferi* by peritoneally recruited neutrophils (a–b: PMN), peritoneally recruited macrophages (c–d: PM) and bone marrow‐derived macrophages (e–f: BMDMs). Raw values are shown in a, c, and e. Uptake in mock‐infected and infected high‐fat diet (HFD) groups normalized to uptake by age‐ and infection‐matched normal diet (ND) controls are shown in b, d, and f. (g–h) *Ex vivo E. coli* killing by BMDMs. g: raw data. h: killing in mock‐infected and infected HFD groups normalized to killing by age‐ and infection‐matched ND controls. For all experiments, neutrophils and macrophages were obtained from male C3H/HeN mice 1 and 8 weeks after inoculation with bacterial cultivation medium alone (mock) or 1 × 10^4^ GCB726 (infected). *N* ≥ 9, 12, and 9 mice per diet and infection group at each time point for PMN, PM, and BMDM experiments, respectively. Summary values are mean ± standard error of the mean (*SEM*). * indicates *p* < 0.05 HFD versus ND within infection group and time point. † indicates *p* < 0.05 infected versus mock within diet group and time point. # indicates *p* < 0.05 8 versus 1 week within diet and infection group. ^ indicates *p* < 0.05 versus ND mock 1 week

In DIO, neutrophilia in response to infection was attenuated and delayed (Figure 3b), and many serum cytokines that were not normally upregulated in response to *B. burgdorferi* infection were cytokines regulating neutrophil production, maturation, survival, recruitment, and activation (Figure 4c–g) (Bugl, Wirths, Müller, Radsak, & Kopp, 2012; Nauseef & Borregaard, 2014). This prompted us to investigate whether DIO affected neutrophil function—specifically *B. burgdorferi* uptake—as well as bacterial uptake by macrophages. Although neutrophil recruitment in DIO mice was not impaired (Figure S6a), complement‐dependent uptake of *B. burgdorferi* (Figure 5a–b) and killing of a control bacterium (Escherichia coli) (Figure S6b) by peritoneally recruited neutrophils from both mock‐ and *B. burgdorferi*‐infected mice were inhibited in DIO. Therefore, DIO alone suppressed neutrophil *B. burgdorferi* uptake and *E. coli* killing. Importantly, DIO‐dependent inhibition of neutrophil *B. burgdorferi* uptake was not affected by mouse infection status at 1 week, but was more pronounced by 8 weeks post‐infection (Figure 5b). Thus, prolonged *B. burgdorferi* infection in the context of DIO synergistically inhibited neutrophil function.

We also investigated the effects of DIO on *B. burgdorferi* uptake and *E. coli* killing by PMs and bone marrow‐derived macrophages (BMDMs). Unlike for neutrophils, DIO alone did not inhibit complement‐dependent *B. burgdorferi* uptake or *E. coli* killing by PMs (Figures 5c–d and S6c) or BMDMs (Figure 5e–h). However, when PMs and BMDMs were obtained from infected HFD mice, *B. burgdorferi* uptake by PMs and BMDMs was impaired (Figure 5c–f), as was *E. coli* killing by BMDMs (Figure 5g–h) but not PMs (Figure S6c). Infection‐dependent suppression of *B. burgdorferi* uptake was observed after both 1 and 8 weeks of infection for PMs (Figure 5c–d), but like neutrophils, was not observed until late in infection for BMDMs (Figure 5e–f). Therefore, *B. burgdorferi* infection and DIO cooperatively suppressed macrophage bacterial uptake. Collectively, these data indicated that although *B. burgdorferi* infection alone did not inhibit neutrophil and macrophage function, DIO and *B. burgdorferi* infection together synergistically and cooperatively suppressed neutrophil and macrophage function, respectively. Furthermore, this effect tended to be greater with longer duration of *B. burgdorferi* infection.

### 2.7 Infection‐dependent global suppression of macrophage cytokine production in response to B. burgdorferi challenge in DIO

**Figure 6:**
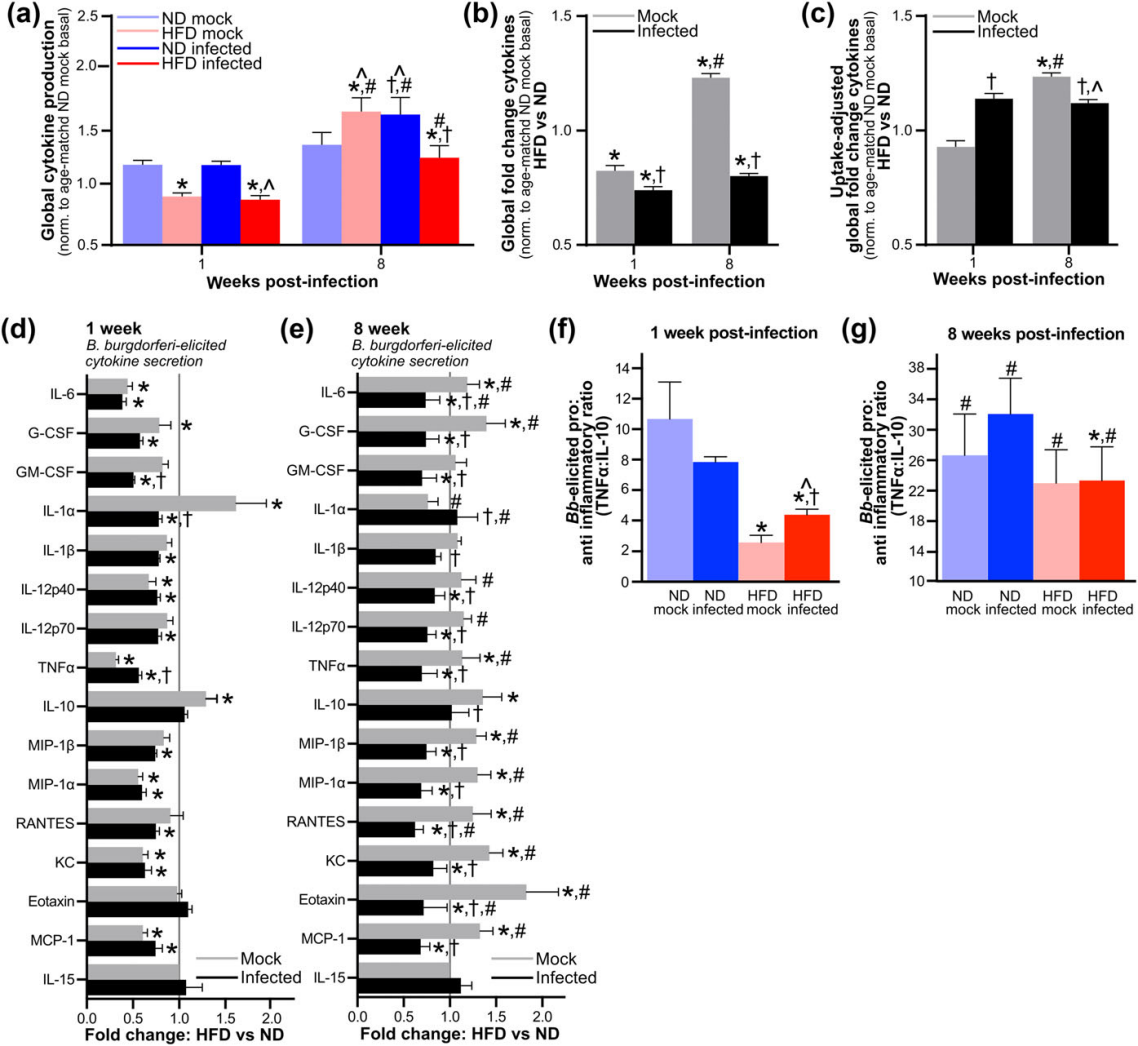
Infection‐dependent global suppression of macrophage cytokine production in response to *Borrelia burgdorferi* challenge in diet‐induced obesity (DIO). Cytokines produced *ex vivo* by peritoneally recruited macrophages from male C3H/HeN mice 1 and 8 weeks after mice were inoculated with cultivation medium alone (mock) or 1 × 10^4^ GCB726 (infected). (a) Global induction of all cytokines by co‐incubation with *B. burgdorferi*, normalized to basal cytokine production by unstimulated PMs from age‐matched normal diet (ND) mock‐infected mice. (b–c) Global high‐fat diet (HFD):ND fold‐difference/cytokine for all cytokines, unadjusted (b) and adjusted (c) for differences in *B. burgdorferi* uptake efficiency among groups (Figure 5c). (d–e) HFD:ND fold‐differences for individual cytokines at 1 (d) and 8 (e) weeks. (f–g) Pro‐inflammatory: anti‐inflammatory (tumor necrosis factor [TNF]α:interleukin [IL]‐10) cytokine ratios for individual mice at 1 (f) and 8 (g) weeks post‐inoculation. Raw data for all panels are provided in Figure S7. *N* ≥ 12 mice per diet group and time point. Data summaries: mean ± standard error of the mean (*SEM*). * indicates *p* < 0.05 HFD versus ND within infection group and time point. † indicates *p* < 0.05 infected versus mock. # indicates *p* < 0.05 8 versus 1 week within infection group. ^ indicates *p* < 0.05 versus age‐matched ND mock group

Finally, we determined if the ability of macrophages to produce cytokines in response to *B. burgdorferi* challenge *ex vivo* was also cooperatively suppressed by *B. burgdorferi* infection and DIO. After 1 week of *B. burgdorferi* infection, global *B. burgdorferi*‐elicited cytokine production by PMs from both mock and infected HFD groups was significantly impaired compared to ND groups (Figure 6a–b). However, after 8 weeks of infection, DIO and *B. burgdorferi* infection alone independently enhanced *B. burgdorferi*‐responsive cytokine production compared to age‐matched ND mock controls, whereas DIO and *B. burgdorferi* infection together suppressed this response (Figure 6a–b). This effect was primarily due to reduced bacterial uptake efficiency in macrophages from infected HFD mice, because *B. burgdorferi*‐elicited global cytokine production in these cells was not impaired compared to ND controls after adjustment for differences in uptake efficiency (Figure 6c). At 1 week post‐inoculation, production of 44% and 81% of individual cytokines was attenuated in HFD compared to ND groups for mock and *B. burgdorferi*‐infected mice, respectively (Figure 6d). By contrast, at 8 weeks post‐inoculation, 0% of individual cytokines were attenuated in HFD versus ND groups for mock‐infected animals, whereas in *B. burgdorferi*‐infected mice production of 75% of cytokines was attenuated in HFD compared to ND animals (Figure 6e). As for serum cytokines (Figure 4i), cytokine production by macrophages from infected HFD mice at both 1 and 8 weeks post‐infection was significantly less pro‐inflammatory than cytokine production by macrophages from infected ND mice (Figure 6f–g), due to reduced TNF‐α production, but not increased IL‐10 secretion (Figure 6d–e). Therefore, *B. burgdorferi* infection and DIO cooperatively suppressed macrophage cytokine production in response to *B. burgdorferi*, and this effect became more pronounced as infection progressed.

## 3 DISCUSSION

Here, we report that cooperative and synergistic suppression of innate immune responses by DIO and *B. burgdorferi* infection together progressively impairs *B. burgdorferi* clearance from tissues and increases the severity of Lyme carditis. We found recently that severe obesity‐independent hyperglycemia is also associated with impaired *B. burgdorferi* tissue clearance and uptake by neutrophils (Javid et al*.*, 2016). However, unlike the results reported here, obesity‐independent hyperglycemia does not increase the severity of inflammatory pathology in *B. burgdorferi* infection and does not act synergistically or cooperatively to suppress innate immune responses (Javid et al*.*, 2016). This, together with our observation that DIO was associated with only mild hyperglycemia, suggest that DIO exerts distinct and more severe, wide‐ranging effects on the outcomes of *B. burgdorferi* infection and host immune responses than hyperglycemia alone.

In humans, obesity is associated with disruption of host immune responses and increased morbidity and mortality due to infection with a range of viruses and bacteria (Hegde & Dhurandhar, 2013; Dhurandhar et al*.*, 2015). DIO is the mouse model of obesity that is thought to most closely approximate human obesity and metabolic syndrome (Hegde & Dhurandhar, 2013). However, the effects of DIO on infection susceptibility and immune responses to bacterial infection in mouse DIO models are surprisingly under‐investigated. Our results are consistent with previous reports of the effects of DIO on infection with S. aureus and the periodontitis‐associated bacterium *P. gingivalis*, which found increased bacterial burden and disease pathology in DIO, suppressed neutrophil uptake and killing of S. aureus, and blunted macrophage production of pro‐inflammatory cytokines in response to *P. gingivalis* (Amar et al*.*, 2007; Yano et al*.*, 2012). Our results also indicate that bacterial infection and DIO together synergistically and cooperatively suppress neutrophil‐ and macrophage‐based responses at later infection stages than the early acute phase investigated in previous studies and that DIO and *B. burgdorferi* infection together can suppress macrophage killing of an unrelated bacterium. These observations are particularly intriguing in light of recent evidence that *B. burgdorferi* infection in normal weight mice suppresses long‐lived antibody responses to itself and antiviral vaccination (Elsner, Hastey, Olsen, & Baumgarth, 2015). Our results indicate that *B. burgdorferi* infection can also suppress non‐adaptive immunity, at least in the context of DIO, and that innate immune responses play an ongoing role in controlling *B. burgdorferi* burden as infection progresses. They also suggest the possibility that immune responses to unrelated bacteria may be disrupted in obese *B. burgdorferi*‐infected hosts.

The major phenotypic outcomes of *B. burgdorferi* infection in DIO in our studies were elevated bacterial DNA burden in heart and other tissues, increased carditis severity in groups where heart burden was elevated, and deficient neutrophil and macrophage *B. burgdorferi* uptake. *B. burgdorferi*‐responsive macrophage cytokine production was attenuated and less pro‐inflammatory at late infection stages, even after adjustment for differences in bacterial uptake efficiency; however, the primary underlying deficiency in cytokine production was likely impaired bacterial internalization in infected mice with DIO. Increased *B. burgdorferi* burden in heart accompanied by more severe carditis pathology is relatively atypical in studies investigating the contributions of different immune cell types and pathways to *B. burgdorferi* infection and pathogenesis. It has previously been observed for mice deficient in iNKT cells, IFN‐γ receptor, CD14, CR3, and macrophage‐specific p38MAPK (Benhnia et al*.*, 2005; Olson et al*.*, 2009; Kelly L. Hawley, Olson, et al., 2012a; Kelly Hawley, Navasa, et al., 2012b). Complement‐dependent *B. burgdorferi* uptake, which was impaired in our studies, is dependent on CR3 and CD14 (Cinco, Murgia, Presani, & Perticarari, 1997; Garcia, Murgia, & Cinco, 2005; Shin et al*.*, 2009; Kelly L. Hawley, Olson, et al., 2012a; Hawley, Martín‐Ruiz, Iglesias‐Pedraz, Berwin, & Anguita, 2013). CD14 plays a complex, possibly modulatory and/or threshold‐sensing role in inflammatory responses in DIO, and is critical for the response to LPS, which is systemically elevated in DIO due to intestinal barrier disruption (Cani et al*.*, 2007; Fernández‐Real et al*.*, 2011). CR3 and CD14, together with p38MAPK, are also part of a pathway that restrains *B. burgdorferi*‐responsive inflammatory cytokine production in macrophages (Sahay et al*.*, 2009; Kelly L. Hawley, Olson, et al., 2012a; Kelly Hawley, Navasa, et al., 2012b; Hawley et al*.*, 2013). Finally, iNKT cells are also progressively depleted as obesity develops in response to HFD (Lynch et al*.*, 2012). The possibilities that the CR3/CD14/p38MAPK pathway and/or iNKT cell function may be disrupted in infected mice with DIO warrant further investigation.

Two unanticipated results of our studies were that *B. burgdorferi* infection induced weight gain at the same rate as HFD feeding and that there was considerable sexual dimorphism in the effects of DIO on *B. burgdorferi* clearance. One of several cytokines that were downregulated upon *B. burgdorferi* infection under normal dietary conditions was IL‐1β, which is an important regulator of MAPK signaling, energy homeostasis, and obesity (Jin et al*.*, 2013). Changes in gut microbiota composition as well as certain viral and bacterial infections have been associated with obesity, although the direction of causality in these associations remains undefined (Hegde & Dhurandhar, 2013; Rosenbaum et al*.*, 2015; Dhurandhar et al*.*, 2015). It is possible that weight gain in *B. burgdorferi*‐infected animals was due to events such as IL‐1β downregulation or a shift in their microbiota, but it is equally possible that factors like reduced physical activity due to infection‐associated discomfort or fatigue contributed to this phenotype.

Innate and adaptive immune responses are sexually dimorphic in humans, mice, and other animals, with female animals exhibiting more robust adaptive immunity, and innate immunity being more prominent in male animals; this results in sexually dimorphic outcomes in certain infections, as well as in DIO (Markle & Fish, 2014; Singer et al*.*, 2015). Sexually dimorphic outcomes of *B. burgdorferi* infection have been reported in the clinical and epidemiological literature (Bennet & Berglund, 2002; Bennet, Stjernberg, & Berglund, 2007; Schwarzwalder, Schneider, Lydecker, & Aucott, 2010; Forrester et al*.*, 2014), but not in mouse studies. A number of sexually dimorphic innate immune mechanisms that are also important for responses to *B. burgdorferi* infection and metabolic regulation could contribute to the prominent effect of DIO on bacterial DNA burden in female mice. These include macrophage activation and production of inflammatory cytokines, neutrophil activation, myelopoiesis, and production of iNKT cells (Weis & Bockenstedt, 2010; Lynch et al*.*, 2012; Cervantes et al*.*, 2014; Petnicki‐Ocwieja & Kern, 2014; Markle & Fish, 2014; Singer et al*.*, 2015).

Finally, we also found that impairment of *B. burgdorferi* clearance in DIO was TLR4‐dependent, especially in brain and liver, that in mice are typically only infected in animals with severe combined immunodeficiency (Schaible et al*.*, 1990; Barthold, Sidman, & Smith, 1992). TLR4 senses dietary lipids and LPS, and liver and brain are major sites regulating TLR4‐dependent induction of systemic inflammatory responses in DIO (Jin et al*.*, 2013; Jia et al*.*, 2014; Morari et al*.*, 2014). *B. burgdorferi* infection in young mice under normal dietary conditions is not controlled by TLR4 but by TLR2, which also senses dietary lipids (Weis & Bockenstedt, 2010; Jin et al*.*, 2013; Cervantes et al*.*, 2014; Petnicki‐Ocwieja & Kern, 2014). However, macrophages from TLR4‐deficient C3H/HeJ mice produce significantly less TNF‐α than those from C3H/HeN animals, the TLR4 agonist LPS suppresses *B. burgdorferi*‐responsive TNF‐α production by human blood cells, and *B. burgdorferi* exposure attenuates macrophage TNF‐α production in response to LPS in a TLR2‐dependent fashion (Diterich, Härter, Hassler, Wendel, & Hartung, 2001; Diterich, Rauter, Kirschning, & Hartung, 2003; Glickstein & Coburn, 2006). Thus, TLR4 clearly modulates immune responses to *B. burgdorferi*, even if these responses are primarily TLR2‐dependent. Prolonged exposure to TLR4 and TLR2 agonists, as well as TNF‐α, can induce macrophage hyporesponsiveness to subsequent challenge with TLR4 and TLR2 agonists, a phenomenon referred to as tolerance or cross‐tolerance (Park, Park‐Min, Chen, Hu, & Ivashkiv, 2011; Seeley & Ghosh, 2013). Thus, TLR4 deficiency could promote *B. burgdorferi* clearance in DIO by a number of possible mechanisms, such as limiting systemic inflammatory responses that impair responsiveness to pathogens and/or by limiting macrophage cross‐tolerance to prolonged stimulation by dietary lipids, *B. burgdorferi* agonists or systemic LPS in DIO, or TNF‐α associated with HFD‐ and *B. burgdorferi*‐induced inflammation. It is also possible that the CR3/CD14/p38MAPK pathway that restrains inflammatory responses to *B. burgdorferi* could contribute to macrophage hyporesponsiveness under these conditions, via its contributions to LPS/TLR4 signaling.

The most important conclusion of the studies reported here is that *B. burgdorferi* infection in the context of DIO suppresses innate immunity. It will be crucial to determine if obesity and/or consumption of diets high in fat affect the outcomes of infection with this emerging pathogen in human populations where obesity is widespread, if *B. burgdorferi* infection is a risk factor for human obesity and if suppression of innate immunity in *B. burgdorferi*‐infected hosts who are obese or consume HFDs increases morbidity and outcomes of other infections.

## 4 EXPERIMENTAL PROCEDURES

### 4.1 Ethics statements

This study was carried out in accordance with the policies of The Canadian Council on Animal Care. Animal work was performed under University of Toronto Animal Care Protocol 010430. All authors, except G. P. W., have no competing interests to declare. G. P. W. reports receiving research grants from Immunetics, Inc., Institute for Systems Biology, Rarecyte, Inc., and bioMérieux SA. G. P. W. owns equity in Abbott, has been an expert witness in malpractice cases involving Lyme disease, and is an unpaid board member of the American Lyme Disease Foundation.

### 4.2 Animals

Animals were housed in environmentally enriched pathogen‐free conditions with *ad libidum* access to water and food in groups of 2–3 per cage during dietary preconditioning (8 weeks for C57BL/6 and 12–14 weeks for C3H mice) and transferred to appropriate Level 2 animal containment quarters within the same facility 1 week before infection. Anesthesia for all experiments was performed using 2% isoflurane (Baxter Corporation, Halifax, NS, Canada), or 10 mg/kg xylazine (MTC Pharmaceuticals, Cambridge, ON, Canada) plus 200 mg/kg ketamine hydrochloride (Rogar/STB, Montréal, QC, Canada). Animals were euthanized by cervical dislocation (anesthetized animals) or CO_2_ asphyxiation (non‐anesthetized animals). Male C57BL/6NCrl and male and female C3H/HeNCrl mice were obtained from Charles River (Montréal, QC, Canada). C3H/HeJCrl male mice were obtained from Jackson Laboratory (Bar Harbor, ME, USA).

### 4.3 Dietary conditioning

As summarized in Figure S1a, age‐, sex‐, and strain‐matched 4‐ to 5‐week old mice were randomly assigned to experimental groups and preconditioned on standard chow (ND or HFD: 60% kcal from fat) for the duration of experiments (12–20 weeks). Unless otherwise indicated in legends, each experimental and diet group included ≥9 mice. For ND (Tekland 2018 Rodent Chow, Harlan Laboratories, Mississauga, ON, Canada), 20.1%, 69.8%, and 10.2% of kcal derived from protein, carbohydrate, and fat, respectively. For HFD (Adjusted Calories Diet (60/Fat) TD.06414, Harlan Laboratories), 18.4%, 21.3%, and 60.3% of kcal derived from protein, carbohydrate, and fat, respectively.

### 4.4 Body weight and plasma glucose measurements

Body weight and peripheral non‐fasting plasma glucose concentrations were monitored throughout study duration as described (Javid et al*.*, 2016). Mice were considered obese if weight of HFD fed animals was 25% greater than age‐matched controls and hyperglycemic when blood glucose was greater than 13 mmol/L (Birk et al*.*, 2014).

### 4.5 B. burgdorferi strains and infections

Unless otherwise indicated in figure legends, all infections were performed with male C3H/HeN mice subcutaneously needle‐inoculated with 1 × 10^4^ *B. burgdorferi* GCB726 at the lumbar dorsal midline or cultivation medium alone (mock). Studies were conducted with low passage *B. burgdorferi* strains GCB726 (B31 5A4 NP1, *ospC*‐type A), N40 (*ospC‐*type E), 297 (*ospC*‐type K), and BL214 (*ospC*‐type B) (Wormser et al*.*, 2008; Moriarty et al*.*, 2008; Petzke & Schwartz, 2015). *B. burgdorferi* freshly inoculated from frozen stocks were cultivated in BSK‐II medium prepared in‐house (Barbour, 1984) containing 6% rabbit serum (Cedarlane Laboratories Ltd, Burlington, ON, Canada) and 100 μg/ml gentamycin (GCB726 only: Bioshop Canada, Burlington, ON), and incubated to log phase at 36°C, 1.5% CO_2_.

### 4.6 Tissue harvesting

Blood for complete blood count (CBC) and serum cytokine analysis was collected from anesthetized animals via cardiac puncture with 1 ml 25G syringes coated with 20 μl 100 U/ml heparin or 4% sodium citrate (Sigma Canada, Oakville, ON). All other tissues (bladder, brain, ear, heart, knee joint (patella), liver, lung, tibiotarsal joint, and skin) were harvested immediately post‐euthanasia. Tissues for histological analysis were placed in 10% buffered formalin (Sigma), which was replaced within 24 hr with fresh formalin. Tissues for qPCR measurement of *flaB* copy number were quick‐frozen on dry ice and stored at −80°C until processing. Tissues for cultivation of viable bacteria were immediately placed in cultivation medium. BMDMs and peritoneally recruited neutrophils and macrophages were obtained in separate experiments from those used for CBC and serum cytokine analysis, histology, cultivation of viable bacteria, and qPCR analysis, as described below.

### 4.7 Isolation of peritoneally recruited neutrophils and macrophages

As described (Javid et al*.*, 2016), peritoneally recruited neutrophils were obtained by peritoneal lavage 2–3 hr after intraperitoneal injection with 1 ml of 5 mM sodium periodate. Neutrophils were counted using a Z1 Coulter particle counter (Beckman Coulter, Fullerton, CA, USA). PMs were obtained by peritoneal lavage 24 hr after intraperitoneal injection of 1 ml of filter‐sterilized 1% brewer's thioglycollate (Sigma). Peritoneal lavage in euthanized mice cleaned with 70% ethanol was performed by intraperitoneal injection of 8–10 ml pre‐warmed 1X dPBS−/− without calcium chloride and magnesium chloride (Sigma), incubation for 1–2 min, and collection of peritoneal fluid using transfer pipettes via a ventral midline incision. If no color change occurred, a second lavage with 1–3 ml dPBS−/− was performed. Sample purity was measured by Shandon Kwik‐Diff stain (Fisher Scientific, Nepean, ON, Canada) according to manufacturer's instructions; purity in all samples was >90%. Macrophages were enumerated using a hemocytometer.

### 4.8 Bone marrow monocyte isolation and macrophage differentiation

Tibias and femurs were excised and cleaned following field sterilization with 70% ethanol. Epiphyses were removed, and bone marrow was flushed using 5 ml of nucleoside‐free αMEM containing L‐glutamine (Life Technologies, Toronto, ON, Canada). Bone marrow from the long bones of each mouse was homogenized with a 20G needle, and single‐cell suspensions were centrifuged for 5 min at 370 × *g*, resuspended in 12 ml of αMEM containing 10% heat inactivated fetal bovine serum (FBS) and 1% penicillin/streptomycin (Life Technologies), plated in a T75 tissue culture flask, and incubated overnight at 37°C 5% CO_2_. Non‐adherent cells (monocytes) were plated in an uncoated 10‐cm bacteriological plate, and 1 μg/ml M‐CSF was added to each dish on the day of plating and 48 hr after plating. Differentiated macrophages were collected for experiments 48 hr after the last M‐CSF addition.

### 4.9 *B. burgdorferi* cultivation from tissues

Tissues (~3 mm^2^) or 100 μl blood from infected mice were incubated in complete BSK‐II medium supplemented with 100 μg/ml gentamycin (GCB726 only), 20 μg/ml phosphomycin (Sigma), 50 μg/ml rifampicin, and 2.5 μg/ml amphotericin (Bioshop) for 1–4 weeks. Following incubation, tissues were identified as positive or negative based on the presence or absence of *Borrelia* detected in media by dark‐field microscopy. One piece of each tissue was cultivated in a single (non‐replicate) culture for each sample.

### 4.10 Quantitative PCR measurement of bacterial DNA copy number in tissues

Isolation of total DNA from tissues using Qiagen DNeasy kits (Toronto, ON, Canada), qPCR measurement of *flaB* DNA copy number, and normalization of copy number to total DNA in extracted samples were performed as described (Moriarty et al*.*, 2012; Javid et al*.*, 2016). Duplicate plasmid DNA (pTM222) standards containing 10^1^–10^6^ copies of the *flaB* amplification template were run alongside sextuplicate reactions for each tissue‐extracted DNA sample, and standard curves calculated for each plate were used to calculate tissue sample *flaB* copy number. Mean copy number values calculated from the six technical replicates for each sample were used for subsequent analyses. Each plate contained negative control wells (DNeasy kit elution buffer AE). Runs with *R* ^2^ values lower than 0.85 were repeated.

For qPCR analysis of tissue colonization, each tissue from each mouse was designated as negative (0%) if the average copy number/sample was ≤1. Samples with an average *flaB* copy >1 were designated as positive (100%). The percentage of tissues/mouse, which were qPCR positive, was calculated by averaging the 0% and 100% values obtained for each tissue. To measure copy number in tissues and control for differences in DNA extraction efficiencies, average copy number for each sample was normalized to the concentration of total DNA in each sample.

### 4.11 Carditis measurement

Scoring of inflammation by counting nuclei in five 100 mm^2^ regions of interest in 2–3 hematoxylin‐ and eosin‐stained sagittal heart sections was performed as described (Diebold et al*.*, 1995; Javid et al*.*, 2016). Numbers of nuclei were enumerated using a counting grid, and the average number of nuclei/region of interest in each section was calculated. Nuclei were counted in each atrium and ventricle and the heart apex. The majority of tissue included in each region of interest was derived from the myocardium.

### 4.12 Complete blood count and serum cytokine analysis

CBC analysis of blood anti‐coagulated with sodium citrate was performed by an IDEXX veterinary reference lab (Markham, ON, Canada), using a Sysmex Hematology Analyzer Model XT2000V (Mississauga, ON, Canada). Cytokine analysis was performed using mouse sera or supernatants from PMs using a Bio‐Plex Pro Mouse Cytokine 23‐plex kit (Bio‐Rad Laboratories, Mississauga, ON, Canada) by the Analytical Facility for Bioactive Molecules (Sick Kids Hospital, Toronto, ON, Canada). PM supernatants for cytokine analysis were obtained from the same experiments in which *ex vivo B. burgdorferi* uptake by PMs was measured, as described below. Basal cytokine production was measured in the same experiments for PMs incubated without *B. burgdorferi*. Supernatants were centrifuged at 4,696 × *g* for 20 min then filtered‐sterilized using a 0.20‐μm filter to remove bacteria, and frozen at −80°C until analysis. Bioplex assays were performed in technical duplicate using samples that were thawed only once before analysis.

### 4.13 Neutrophil bacterial uptake assays

Uptake of *E. coli* DH5α cultured overnight at 37°C in LB broth and log phase GCB726 *B. burgdorferi* by peritoneally recruited neutrophils was performed as described (Javid et al*.*, 2016). Bacteria (3 × 10^6^ and 1 × 10^7^ *E. coli* and *B. burgdorferi*, respectively) were complement‐opsonized with 5 μl pre‐immune serum from isogenic ND mice for 30 min at 37°C/5% CO_2_ (*E. coli*) or 36°C/1.5% CO_2_ (*B. burgdorferi*)_._ Complement‐opsonized bacteria were added to 1 × 10^6^ neutrophils in RPMI medium (Sigma) supplemented with 5% FBS at multiplicities of infection (MOIs) of 3:1 and 10:1 for *E. coli* and *B. burgdorferi*, respectively. Bacteria were co‐incubated with neutrophils for 1 hr at 37°C/5% CO_2_ (*E. coli*) or 16 hr at 36°C/1.5% CO_2_ (*B. burgdorferi*). Dilutions of *E. coli* co‐incubations were plated on LB plates incubated for 16 hr at 37°C, followed by counting of colony forming units. Numbers of intact *B. burgdorferi* remaining following neutrophil co‐incubation were counted using a Petroff‐Hauser counting chamber. Numbers of *E. coli* colony forming units and intact *B. burgdorferi* for neutrophil co‐incubations were normalized to numbers of input bacteria mock‐incubated in the absence of neutrophils to determine percentages of bacteria which were killed (*E. coli*) and taken up (*B. burgdorferi*) by neutrophils.

### 4.14 Bacterial uptake assays for peritoneally recruited and bone marrow derived macrophages

Freshly isolated PMs were seeded at 1 × 10^6^ cells/well in six‐well tissue culture plates and allowed to adhere for 2 hr at 37°C/5% CO_2_ before bacterial uptake assays. Differentiated BMDMs were incubated for 10 min in PBS containing 5 mM EDTA, collected by gentle scraping, centrifuged for 10 min at 300 × *g*, resuspended in antibiotic‐free αMEM containing 10% FBS and 1 μg/ml M‐CSF, plated at 1 × 10^6^ cells/well, and incubated for 24 hr at 37°C/5% CO^2^ before bacterial uptake assays. For both PMs and BMDMs, media were replaced with fresh, antibiotic‐free αMEM containing 10% FBS immediately before bacterial uptake assays.

Cultivation and complement‐opsonization of bacteria for macrophage uptake assays were performed as described for neutrophil uptake assays, as was quantification of % survival and % uptake relative to mock‐treated input controls. Bacteria were co‐incubated with 1 × 10^6^ macrophages in 1 ml of antibiotic‐free αMEM containing 10% FBS at 37°C/5% CO_2_. *E. coli* were co‐incubated with macrophages at an MOI of 3:1 for 1 hr. *B. burgdorferi* were co‐incubated with macrophages at an MOI of 10:1 for 2 hr.

### 4.15 Statistical analysis

Statistical analysis was performed using GraphPad Prism v.6.0 graphing and statistical analysis software (GraphPad Software, La Jolla, CA, USA). Specific statistical tests used in each experiment are indicated in figure legends.

## Supporting information

Supporting info item

## References

[cmi12689-bib-0001] Amar, S. , Zhou, Q. , Shaik‐Dasthagirisaheb, Y. , & Leeman, S. (2007). Diet‐induced obesity in mice causes changes in immune responses and bone loss manifested by bacterial challenge. Proceedings of the National Academy of Sciences of the United States of America, 104(51), 20466–20471. doi:10.1073/pnas.0710335105 18077329 10.1073/pnas.0710335105 PMC2154454

[cmi12689-bib-0002] Barbour, A. G. (1984). Isolation and cultivation of Lyme disease spirochetes. The Yale Journal of Biology and Medicine, 57(4), 521–525. 6393604 PMC2589996

[cmi12689-bib-0003] Barthold, S. W. , Cadavid, D. , & Philipp, M. T. (2010). Animal models of borreliosis In Radolf J. D., & Samuels D. S. (Eds.), Borrelia*: Molecular biology, host interaction, and pathogenesis*. (pp. 359–411). Norfolk, UK: Caister Academic Press.

[cmi12689-bib-0004] Barthold, S. W. , Sidman, C. L. , & Smith, A. L. (1992). Lyme borreliosis in genetically resistant and susceptible mice with severe combined immunodeficiency. The American Journal of Tropical Medicine and Hygiene, 47(5), 605–613. 1449201 10.4269/ajtmh.1992.47.605

[cmi12689-bib-0005] Benhnia, M. R. , Wroblewski, D. , Akhtar, M. N. , Patel, R. A. , Lavezzi, W. , Gangloff, S. C. , … Sellati, T. J. (2005). Signaling through CD14 attenuates the inflammatory response to *Borrelia burgdorferi*, the agent of Lyme disease. Journal of Immunology, 174(3), 1539–1548. doi:10.4049/jimmunol.174.3.1539 10.4049/jimmunol.174.3.1539 15661914

[cmi12689-bib-0006] Bennet, L. , & Berglund, J. (2002). Reinfection with Lyme borreliosis: a retrospective follow‐up study in southern Sweden. Scandinavian Journal of Infectious Diseases, 34(3), 183–186. 12030390 10.1080/00365540110080070

[cmi12689-bib-0007] Bennet, L. , Stjernberg, L. , & Berglund, J. (2007). Effect of gender on clinical and epidemiologic features of Lyme borreliosis. Vector Borne and Zoonotic Diseases, 7(1), 34–41. doi:10.1089/vbz.2006.0533 17417955 10.1089/vbz.2006.0533

[cmi12689-bib-0008] Birk, R. Z. , Rubio‐Aliaga, I. , Boekschoten, M. V. , Danino, H. , Muller, M. , & Hannelore, D. (2014). Differential regulation of pancreatic digestive enzymes during chronic high‐fat diet‐induced obesity in C57BL/6J mice, *112*, 154–161. doi:10.1017/S0007114514000816 10.1017/S0007114514000816 24816161

[cmi12689-bib-0009] Bockenstedt, L. K. , Gonzalez, D. G. , Haberman, A. M. , & Belperron, A. A. (2012). Spirochete antigens persist near cartilage after murine Lyme borreliosis therapy. The Journal of Clinical Investigation, 122(7), 2652–2660. doi:10.1172/JCI58813 22728937 10.1172/JCI58813 PMC3386809

[cmi12689-bib-0010] Bugger, H. , & Abel, E. D. (2009). Rodent models of diabetic cardiomyopathy. Disease Models & Mechanisms, 2(9–10), 454–466. doi:10.1242/dmm.001941 19726805 10.1242/dmm.001941

[cmi12689-bib-0011] Bugl, S. , Wirths, S. , Müller, M. R. , Radsak, M. P. , & Kopp, H.‐G. (2012). Current insights into neutrophil homeostasis. Annals of the New York Academy of Sciences, 1266, 171–178. doi:10.1111/j.1749-6632.2012.06607.x 22901268 10.1111/j.1749-6632.2012.06607.x

[cmi12689-bib-0012] Cani, P. D. , Amar, J. , Iglesias, M. A. , Poggi, M. , Knauf, C. , Bastelica, D. , … Burcelin, R. (2007). Metabolic endotoxemia initiates obesity and insulin resistance. Diabetes, 56(7), 1761–1772. doi:10.2337/db06-1491 17456850 10.2337/db06-1491

[cmi12689-bib-0013] Cervantes, J. L. , Hawley, K. L. , Benjamin, S. J. , Weinerman, B. , Luu, S. M. , & Salazar, J. C. (2014). Phagosomal TLR signaling upon *Borrelia burgdorferi* infection. Frontiers in Cellular and Infection Microbiology, 4, 1–12. doi:10.3389/fcimb.2014.00055 24904837 10.3389/fcimb.2014.00055 PMC4033037

[cmi12689-bib-0014] Cinco, M. , Murgia, R. , Presani, G. , & Perticarari, S. (1997). Integrin CR3 mediates the binding of nonspecifically opsonized *Borrelia burgdorferi* to human phagocytes and mammalian cells. Infection and Immunity, 65(11), 4784–4789. 9353065 10.1128/iai.65.11.4784-4789.1997 PMC175686

[cmi12689-bib-0015] Dhurandhar, N. V. , Bailey, D. , & Thomas, D. (2015). Interaction of obesity and infections. Obesity Reviews, 16(12), 1017–1029. doi:10.1111/obr.12320 26354800 10.1111/obr.12320

[cmi12689-bib-0016] Diebold, R. J. , Eis, M. J. , Yin, M. , Ormsby, I. , Boivin, G. P. , Darrow, B. J. , … Doetschman, T. (1995). Early‐onset multifocal inflammation in the transforming growth factor β1‐null mouse is lymphocyte mediated. Proceedings of the National Academy of Sciences of the United States of America, 92(26), 12215–12219. 8618872 10.1073/pnas.92.26.12215 PMC40327

[cmi12689-bib-0017] Diterich, I. , Härter, L. , Hassler, D. , Wendel, A. , & Hartung, T. (2001). Modulation of cytokine release in *ex vivo*‐stimulated blood from borreliosis patients. Infection and Immunity, 69(2), 687–694. doi:10.1128/IAI.69.2.687-694.2001 11159956 10.1128/IAI.69.2.687-694.2001 PMC97940

[cmi12689-bib-0018] Diterich, I. , Rauter, C. , Kirschning, C. J. , & Hartung, T. (2003). *Borrelia burgdorferi*‐induced tolerance as a model of persistence via immunosuppression. Infection and Immunity, 71(7), 3979–3987. doi:10.1128/IAI.71.7.3979-3987.2003 12819085 10.1128/IAI.71.7.3979-3987.2003 PMC162029

[cmi12689-bib-0019] Elsner, R. A. , Hastey, C. J. , Olsen, K. J. , & Baumgarth, N. (2015). Suppression of long‐lived humoral immunity following *Borrelia burgdorferi* infection. PLoS Pathogens, 11(7) e1004976. doi:10.1371/journal.ppat.1004976 10.1371/journal.ppat.1004976 PMC4489802 26136236

[cmi12689-bib-0020] Farnsworth, C. W. , Shehatou, C. T. , Maynard, R. , Nishitani, K. , Kates, S. L. , Zuscik, M. J. , … Mooney, R. A. (2015). A humoral immune defect distinguishes the response to *Staphylococcus aureus* infections in mice with obesity and type 2 diabetes from that in mice with type 1 diabetes. Infection and Immunity, 83(6), 2264–2274. doi:10.1128/IAI.03074-14 25802056 10.1128/IAI.03074-14 PMC4432732

[cmi12689-bib-0021] Fernández‐Real, J. M. , Pérez del Pulgar, S. , Luche, E. , Moreno‐Navarrete, J. M. , Waget, A. , Serino, M. , … Zorzano, A. (2011). CD14 modulates inflammation‐driven insulin resistance. Diabetes, 60(8), 2179–2186. doi:10.2337/db10-1210 21700881 10.2337/db10-1210 PMC3142089

[cmi12689-bib-0022] Finucane, M. M. , Stevens, G. A. , Cowan, M. J. , Danaei, G. , Lin, J. K. , Paciorek, C. J. , … Global Burden of Metabolic Risk Factors of Chronic Diseases Collaborating Group (Body Mass Index) (2011). National, regional, and global trends in body‐mass index since 1980: systematic analysis of health examination surveys and epidemiological studies with 960 country‐years and 9·1 million participants. Lancet, 377(9765), 557–567. doi:10.1016/S0140-6736(10)62037-5 21295846 10.1016/S0140-6736(10)62037-5 PMC4472365

[cmi12689-bib-0023] Forrester, J. D. , Meiman, J. , Mullins, J. , Nelson, R. , Ertel, S.‐H. , Cartter, M. , … Centers for Disease Control and Prevention (CDC) (2014). Notes from the field: update on Lyme carditis, groups at high risk, and frequency of associated sudden cardiac death‐‐United States. MMWR. Morbidity and Mortality Weekly Report, 63(43), 982–983. 25356607 PMC5779475

[cmi12689-bib-0024] Garcia, R. C. , Murgia, R. , & Cinco, M. (2005). Complement receptor 3 binds the *Borrelia burgdorferi* outer surface proteins OspA and OspB in an iC3b‐independent manner. Infection and Immunity, 73(9), 6138–6142. doi:10.1128/IAI.73.9.6138-6142.2005 16113335 10.1128/IAI.73.9.6138-6142.2005 PMC1231105

[cmi12689-bib-0025] Glickstein, L. J. , & Coburn, J. L. (2006). Short report: Association of macrophage inflammatory response and cell death after in vitro *Borrelia burgdorferi* infection with arthritis resistance. The American Journal of Tropical Medicine and Hygiene, 75(5), 964–967. 17123997

[cmi12689-bib-0026] Hawley, K. L. , Martín‐Ruiz, I. , Iglesias‐Pedraz, J. M. , Berwin, B. , & Anguita, J. (2013). CD14 targets complement receptor 3 to lipid rafts during phagocytosis of *Borrelia burgdorferi* . International Journal of Biological Sciences, 9(8), 803–810. doi:10.7150/ijbs.7136 23983613 10.7150/ijbs.7136 PMC3753444

[cmi12689-bib-0027] Hawley, K. L. , Olson, C. M. , Iglesias‐Pedraz, J. M. , Navasa, N. , Cervantes, J. L. , Caimano, M. J. , … Anguita, J. (2012a). CD14 cooperates with complement receptor 3 to mediate MyD88‐independent phagocytosis of *Borrelia burgdorferi* . Proceedings of the National Academy of Sciences of the United States of America, 109(4), 1228–1232. doi:10.1073/pnas.1112078109 22232682 10.1073/pnas.1112078109 PMC3268315

[cmi12689-bib-0028] Hawley, K. , Navasa, N. , Olson, C. M. , Bates, T. C. , Garg, R. , Hedrick, M. N. , … Anguita, J. (2012b). Macrophage p38 mitogen‐activated protein kinase activity regulates invariant natural killer T‐cell responses during *Borrelia burgdorferi* infection. The Journal of Infectious Diseases, 206(2), 283–291. doi:10.1093/infdis/jis332 22551807 10.1093/infdis/jis332 PMC3490691

[cmi12689-bib-0029] Hegde, V. , & Dhurandhar, N. V. (2013). Microbes and obesity‐‐interrelationship between infection, adipose tissue and the immune system. Clinical Microbiology and Infection, 19(4), 314–320. doi:10.1111/1469-0691.12157 23506525 10.1111/1469-0691.12157

[cmi12689-bib-0030] Hodzic, E. , Imai, D. , Feng, S. , & Barthold, S. W. (2014). Resurgence of persisting non‐cultivable *Borrelia burgdorferi* following antibiotic treatment in mice. PloS One, 9(1) e86907. doi:10.1371/journal.pone.0086907 10.1371/journal.pone.0086907 PMC3900665 24466286

[cmi12689-bib-0031] Isogai, E. , Isogai, H. , Kimura, K. , Hayashi, S. , Kubota, T. , Nishikawa, T. , … Fujii, N. (1996). Cytokines in the serum and brain in mice infected with distinct species of Lyme disease *Borrelia* . Microbial Pathogenesis, 21(6), 413–419. doi:10.1006/mpat.1996.0072 8971682 10.1006/mpat.1996.0072

[cmi12689-bib-0032] Javid, A. , Zlotnikov, N. , Pětrošová, H. , Tang, T. T. , Zhang, Y. , Bansal, A. K. , … Moriarty, T. J. (2016). Hyperglycemia impairs neutrophil‐mediated bacterial clearance in mice infected with the Lyme disease pathogen. PloS One, 11(6) e0158019. doi:10.1371/journal.pone.0158019 10.1371/journal.pone.0158019 PMC4920391 27340827

[cmi12689-bib-0033] Jia, L. , Vianna, C. R. , Fukuda, M. , Berglund, E. D. , Liu, C. , Tao, C. , … Elmquist, J. K. (2014). Hepatocyte Toll‐like receptor 4 regulates obesity‐induced inflammation and insulin resistance. Nature Communications, 5, 1–26. doi:10.1371/journal.pone.0086907 10.1038/ncomms4878 PMC4080408 24815961

[cmi12689-bib-0034] Jin, C. , Henao‐Mejia, J. , & Flavell, R. A. (2013). Innate immune receptors: key regulators of metabolic disease progression. Cell Metabolism, 17(6), 873–882. doi:10.1016/j.cmet.2013.05.011 23747246 10.1016/j.cmet.2013.05.011

[cmi12689-bib-0035] Lynch, L. , Nowak, M. , Varghese, B. , Clark, J. , Hogan, A. E. , Toxavidis, V. , … Exley, M. A. (2012). Adipose tissue invariant NKT cells protect against diet‐induced obesity and metabolic disorder through regulatory cytokine production. Immunity, 37(3), 574–587. doi:10.1016/j.immuni.2012.06.016 22981538 10.1016/j.immuni.2012.06.016 PMC4991771

[cmi12689-bib-0036] Markle, J. G. , & Fish, E. N. (2014). SeXX matters in immunity. Trends in Immunology, 35(3), 97–104. doi:10.1016/j.it.2013.10.006 24239225 10.1016/j.it.2013.10.006

[cmi12689-bib-0037] McNelis, J. C. , & Olefsky, J. M. (2014). Macrophages, immunity, and metabolic disease. Immunity, 41(1), 36–48. doi:10.1016/j.immuni.2014.05.010 25035952 10.1016/j.immuni.2014.05.010

[cmi12689-bib-0038] Mead, P. S. (2015). Epidemiology of Lyme disease. Infectious Disease Clinics of North America, 29(2), 187–210. doi:10.1016/j.idc.2015.02.010 25999219 10.1016/j.idc.2015.02.010

[cmi12689-bib-0039] Morari, J. , Anhe, G. F. , Nascimento, L. F. , de Moura, R. F. , Razolli, D. , Solon, C. , … Velloso, L. A. (2014). Fractalkine (CX3CL1) is involved in the early activation of hypothalamic inflammation in experimental obesity. Diabetes, 63(11), 3770–3784. doi:10.2337/db13-1495 24947351 10.2337/db13-1495

[cmi12689-bib-0040] Moriarty, T. J. , Norman, M. U. , Colarusso, P. , Bankhead, T. , Kubes, P. , & Chaconas, G. (2008). Real‐time high resolution 3D imaging of the Lyme disease spirochete adhering to and escaping from the vasculature of a living host. PLoS Pathogens, 4(6) e1000090. doi:10.1371/journal.ppat.1000090 10.1371/journal.ppat.1000090 PMC2408724 18566656

[cmi12689-bib-0041] Moriarty, T. J. , Shi, M. , Lin, Y.‐P. , Ebady, R. , Zhou, H. , Odisho, T. , … Chaconas, G. (2012). Vascular binding of a pathogen under shear force through mechanistically distinct sequential interactions with host macromolecules. Molecular Microbiology, 86(5), 1116–1131. doi:10.1111/mmi.12045 23095033 10.1111/mmi.12045 PMC3508296

[cmi12689-bib-0042] Nauseef, W. M. , & Borregaard, N. (2014). Neutrophils at work. Nature Immunology, 15(7), 602–611. doi:10.1038/ni.2921 24940954 10.1038/ni.2921

[cmi12689-bib-0043] Olson, C. M. , Bates, T. C. , Izadi, H. , Radolf, J. D. , Huber, S. A. , Boyson, J. E. , & Anguita, J. (2009). Local production of IFN‐γ by invariant NKT cells modulates acute Lyme carditis. Journal of Immunology, 182(6), 3728–3734. doi:10.4049/jimmunol.0804111 10.4049/jimmunol.0804111 PMC2679988 19265151

[cmi12689-bib-0044] Park, S. H. , Park‐Min, K.‐H. , Chen, J. , Hu, X. , & Ivashkiv, L. B. (2011). Tumor necrosis factor induces GSK3 kinase‐mediated cross‐tolerance to endotoxin in macrophages. Nature Immunology, 12(7), 607–615. doi:10.1038/ni.2043 21602809 10.1038/ni.2043 PMC3258532

[cmi12689-bib-0045] Petnicki‐Ocwieja, T. , & Kern, A. (2014). Mechanisms of *Borrelia burgdorferi* internalization and intracellular innate immune signaling. Frontiers in Cellular and Infection Microbiology, 4, 1–7. doi:10.3389/fcimb.2014.00175 25566512 10.3389/fcimb.2014.00175 PMC4266086

[cmi12689-bib-0046] Petzke, M. , & Schwartz, I. (2015). *Borrelia burgdorferi* pathogenesis and the immune response. Clinics in Laboratory Medicine, 35(4), 745–764. doi:10.1016/j.cll.2015.07.004 26593255 10.1016/j.cll.2015.07.004

[cmi12689-bib-0047] Rosenbaum, M. , Knight, R. , & Leibel, R. L. (2015). The gut microbiota in human energy homeostasis and obesity. Trends in Endocrinology and Metabolism, 493–501. doi:10.1016/j.tem.2015.07.002 26257300 10.1016/j.tem.2015.07.002 PMC4862197

[cmi12689-bib-0048] Sahay, B. , Patsey, R. L. , Eggers, C. H. , Salazar, J. C. , Radolf, J. D. , & Sellati, T. J. (2009). CD14 signaling restrains chronic inflammation through induction of p38‐MAPK/SOCS‐dependent tolerance. PLoS Pathogens, 5(12) e1000687. doi:10.1371/journal.ppat.1000687 10.1371/journal.ppat.1000687 PMC2781632 20011115

[cmi12689-bib-0049] Schaible, U. E. , Gay, S. , Museteanu, C. , Kramer, M. D. , Zimmer, G. , Eichmann, K. , … Simon, M. M. (1990). Lyme borreliosis in the severe combined immunodeficiency (scid) mouse manifests predominantly in the joints, heart, and liver. The American Journal of Pathology, 137(4), 811–820. 2221014 PMC1877559

[cmi12689-bib-0050] Schwarzwalder, A. , Schneider, M. F. , Lydecker, A. , & Aucott, J. N. (2010). Sex differences in the clinical and serologic presentation of early Lyme disease: Results from a retrospective review. Gender Medicine, 7(4), 320–329. doi:10.1016/j.genm.2010.08.002 20869632 10.1016/j.genm.2010.08.002

[cmi12689-bib-0051] Seeley, J. J. , & Ghosh, S. (2013). Tolerization of inflammatory gene expression. Cold Spring Harbor Symposia on Quantitative Biology, 78, 69–79. doi:10.1101/sqb.2013.78.020040 25028399 10.1101/sqb.2013.78.020040

[cmi12689-bib-0052] Shin, O. S. , Miller, L. S. , Modlin, R. L. , Akira, S. , Uematsu, S. , & Hu, L. T. (2009). Downstream signals for MyD88‐mediated phagocytosis of *Borrelia burgdorferi* can be initiated by TRIF and are dependent on PI3K. Journal of Immunology, 183(1), 491–498. doi:10.4049/jimmunol.0900724 10.4049/jimmunol.0900724 PMC2772066 19542460

[cmi12689-bib-0053] Singer, K. , Maley, N. , Mergian, T. , DelProposto, J. , Cho, K. W. , Zamarron, B. F. , … Lumeng, C. N. (2015). Differences in hematopoietic stem cells contribute to sexually dimorphic inflammatory responses to high hat diet‐induced obesity. The Journal of Biological Chemistry, 290(21), 13250–13262. doi:10.1074/jbc.M114.634568 25869128 10.1074/jbc.M114.634568 PMC4505578

[cmi12689-bib-0054] Toledo, A. , Monzón, J. D. , Coleman, J. L. , Garcia‐Monco, J. C. , & Benach, J. L. (2015). Hypercholesterolemia and ApoE deficiency result in severe infection with Lyme disease and relapsing‐fever *Borrelia* . Proceedings of the National Academy of Sciences of the United States of America, 112(17), 5491–5496. doi:10.1073/pnas.1502561112 25870274 10.1073/pnas.1502561112 PMC4418910

[cmi12689-bib-0055] Varol, C. , Mildner, A. , & Jung, S. (2015). Macrophages: development and tissue specialization. Annual Review of Immunology, 33, 643–675. doi:10.1146/annurev-immunol-032414-112220 10.1146/annurev-immunol-032414-112220 25861979

[cmi12689-bib-0056] Wang, C.‐Y. , & Liao, J. K. (2012). A mouse model of diet‐induced obesity and insulin resistance. Methods in Molecular Biology, 821, 421–433. doi:10.1007/978-1-61779-430-8_27 22125082 10.1007/978-1-61779-430-8_27 PMC3807094

[cmi12689-bib-0057] Weis, J. , & Bockenstedt, L. (2010). Host response In Radolf J. D., & Samuels D. S. (Eds.), Borrelia*: Molecular biology, host interaction, and pathogenesis*. (pp. 413–442). Norfolk, UK: Caister Academic Press.

[cmi12689-bib-0058] Wormser, G. P. , Brisson, D. , Liveris, D. , Hanincova, K. , Sandigursky, S. , Nowakowski, J. , … Schwartz, I. (2008). *Borrelia burgdorferi* genotype predicts the capacity for hematogenous dissemination during early Lyme disease. The Journal of Infectious Diseases, 198(9), 1358–1364. doi:10.1371/journal.ppat.1000090 18781866 10.1086/592279 PMC2776734

[cmi12689-bib-0059] Yano, H. , Kinoshita, M. , Fujino, K. , Nakashima, M. , Yamamoto, Y. , Miyazaki, H. , … Tanaka, Y. (2012). Insulin treatment directly restores neutrophil phagocytosis and bactericidal activity in diabetic mice and thereby improves surgical site *Staphylococcus aureus* infection. Infection and Immunity, 80(12), 4409–4416. doi:10.1128/IAI.00787-12 23027538 10.1128/IAI.00787-12 PMC3497398

[cmi12689-bib-0060] Zhang, Y. , Li, Q. , Rao, E. , Sun, Y. , Grossmann, M. E. , Morris, R. J. , … Li, B. (2015). Epidermal Fatty Acid binding protein promotes skin inflammation induced by high‐fat diet. Immunity, 42(5), 953–964. doi:10.1016/j.immuni.2015.04.016 25992864 10.1016/j.immuni.2015.04.016 PMC4440244

