# Supplementary material for: Infection with the Lyme disease pathogen suppresses innate immunity in mice with diet‐induced obesity
Source: Cell Microbiol. 2016 Nov 25;19(5):e12689. doi: 10.1111/cmi.12689 (PMC5383418; doi:10.1111/cmi.12689)
Supplement: Supplementary file 1 — Supporting info item [file CMI-19-na-s001.docx]

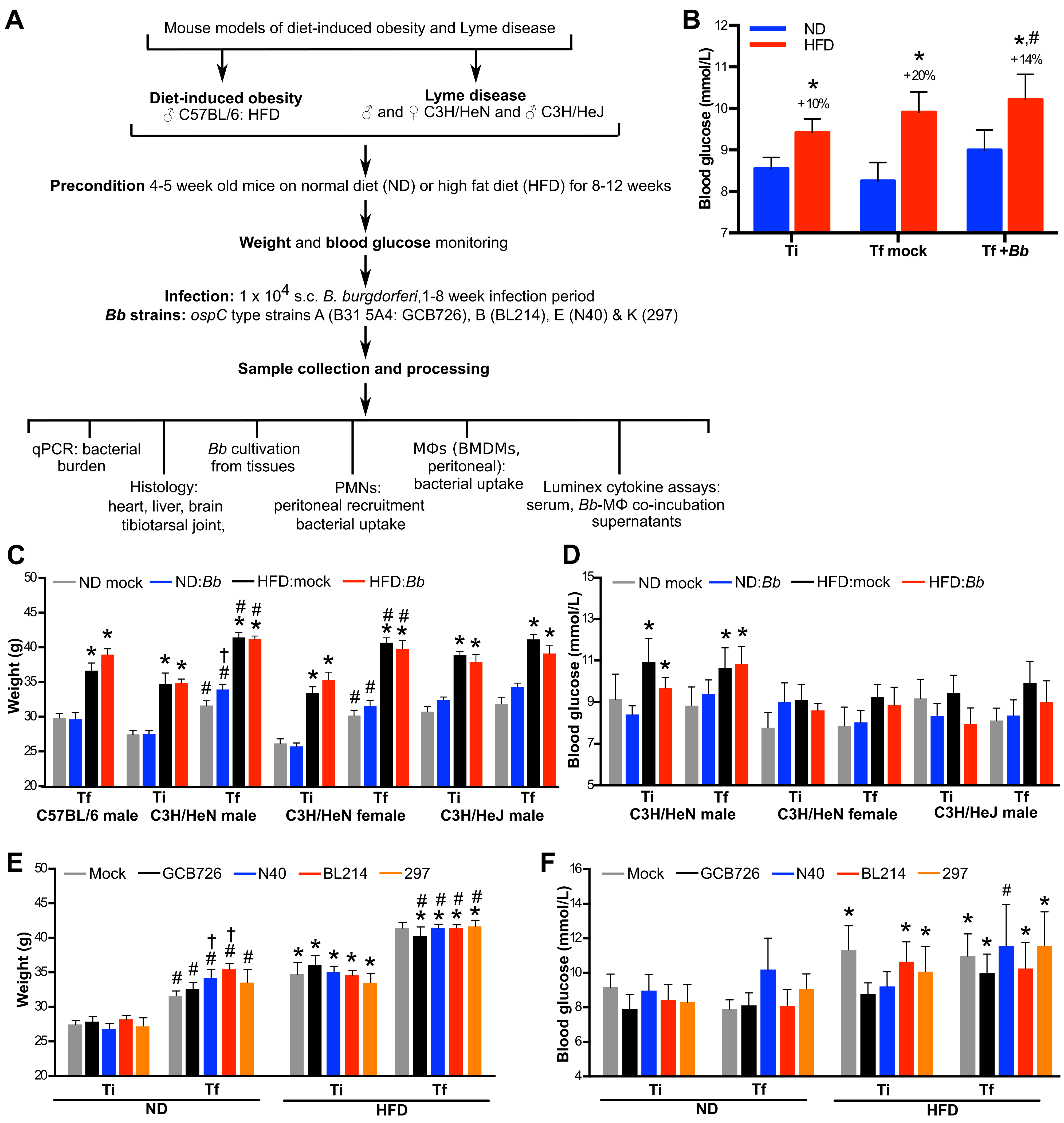
**FIG S1. Body weight and blood glucose levels in reported experiments.** (**A**) Schematic depicting experimental variables and outcomes measured in study (all experiments reported in this paper). Mice were fed standard (ND) or high fat diet (HFD; 60% kcal from fat) for 10 (C57BL/6) or 12 (C3H) weeks followed by 1-8 weeks of *B. burgdorferi* infection. (**B**) Non-fasting blood glucose at time of infection (Ti) and final sacrifice (Tf:) measured for mice infected for 4 weeks with 1 x 10^4^ *B. burgdorferi* (+*Bb*) or vehicle (mock: bacterial cultivation medium alone). Global mean ± 95% confidence intervals are shown for all mouse sexes and strains and bacterial strains analyzed in this study. Values above data columns indicate % increase in blood glucose in mice fed HFD compared to ND. N=28-93 age and sex-matched mice per group. p<0.05 vs ND (*), vs Ti within diet (#). **(C-F)** Mean ±SEM body weight **(C, E)** and non-fasting blood glucose **(D, F)** for indicated mouse strains, sexes and *Borrelia* strains. Mice were sacrificed after 4 weeks of infection. In C and D, values for HeN male mice are pooled from experiments performed with 4 different *B. burgdorferi* strains: GCB726, N40, BL314 and 297. Values for experiments performed with each of these strains are shown in E and F. Blood glucose levels were not measured for BL/6 mice. N=8-40 mice/group (8-11 mice/diet group in each experiment). For C-F, *p<0.05 vs ND within strain and sex group; †p<0.05 vs Tf mock within strain and sex group. #p<0.05 vs Ti within strain and sex group. Statistics (all panels): 2-way ANOVA, Holm-Sidak post-tests.


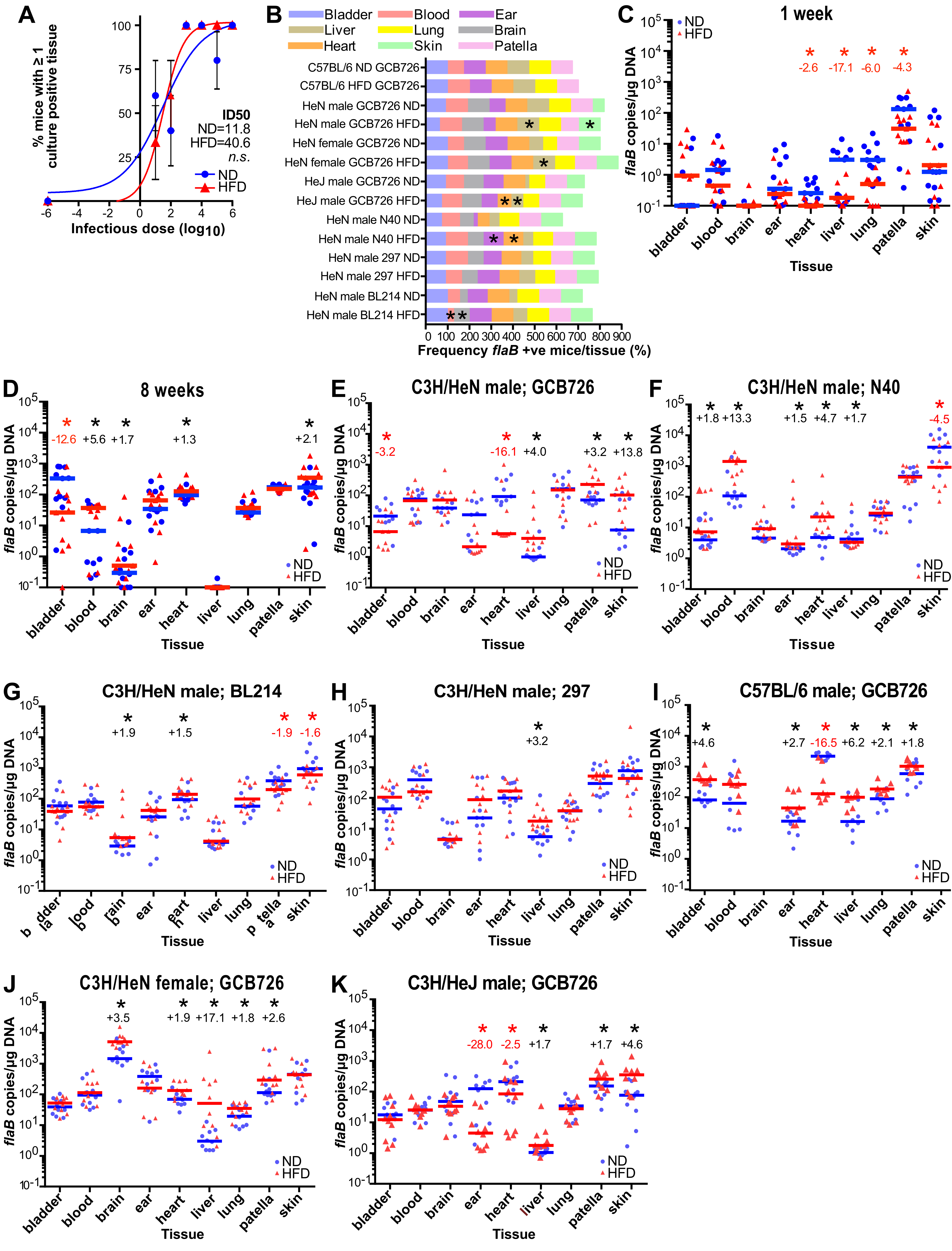


**FIG S2. *B. burgdorferi* infectivity, colonization and bacterial DNA burden in tissues of mouse strains and sexes reported in this study. (A)** Infectious doses (ID50s) calculated by comparing % of mice with at least 1 culture-positive tissue 3 weeks after inoculation of male C3H/HeN mice with indicated doses of GCB726. N=5 mice/diet and dose group. ID50 values calculated from dose-response curves by least squares fit non-linear regression are indicated in the legend. Statistical comparison of ID50s: Extra sum-of-squares F-test (n.s.: no significant difference). **(B)** Frequency of % of *flaB*-positive mice per tissue at 4 weeks post-inoculation with 1 x 10^4^ *B. burgdorferi*. * indicates p<0.05 vs. ND. **(C-D)** *flaB* copy number/µg extracted DNA for male C3H/HeN mice infected with 1 x 10^4^ GCB726 for 1 (C) and 8 (D) weeks. **(E-K)** *flaB* copy number / µg DNA for indicated mouse strains and sexes infected for 4 weeks with 1 x 10^4^ of indicated bacterial strains. In C-K, each data point corresponds to the *flaB* copy number/µg DNA for 1 tissue from 1 mouse. Bars indicate median copy number values for each tissue and diet group. Values above datasets for each tissue indicate significant fold-differences in medians for tissues from mice fed HFD vs ND. N= 9-11 mice/group. Statistics: 2-way ANOVA of log-transformed values with Holm-Sidak post-test; * indicates p<0.05 HFD vs. ND.


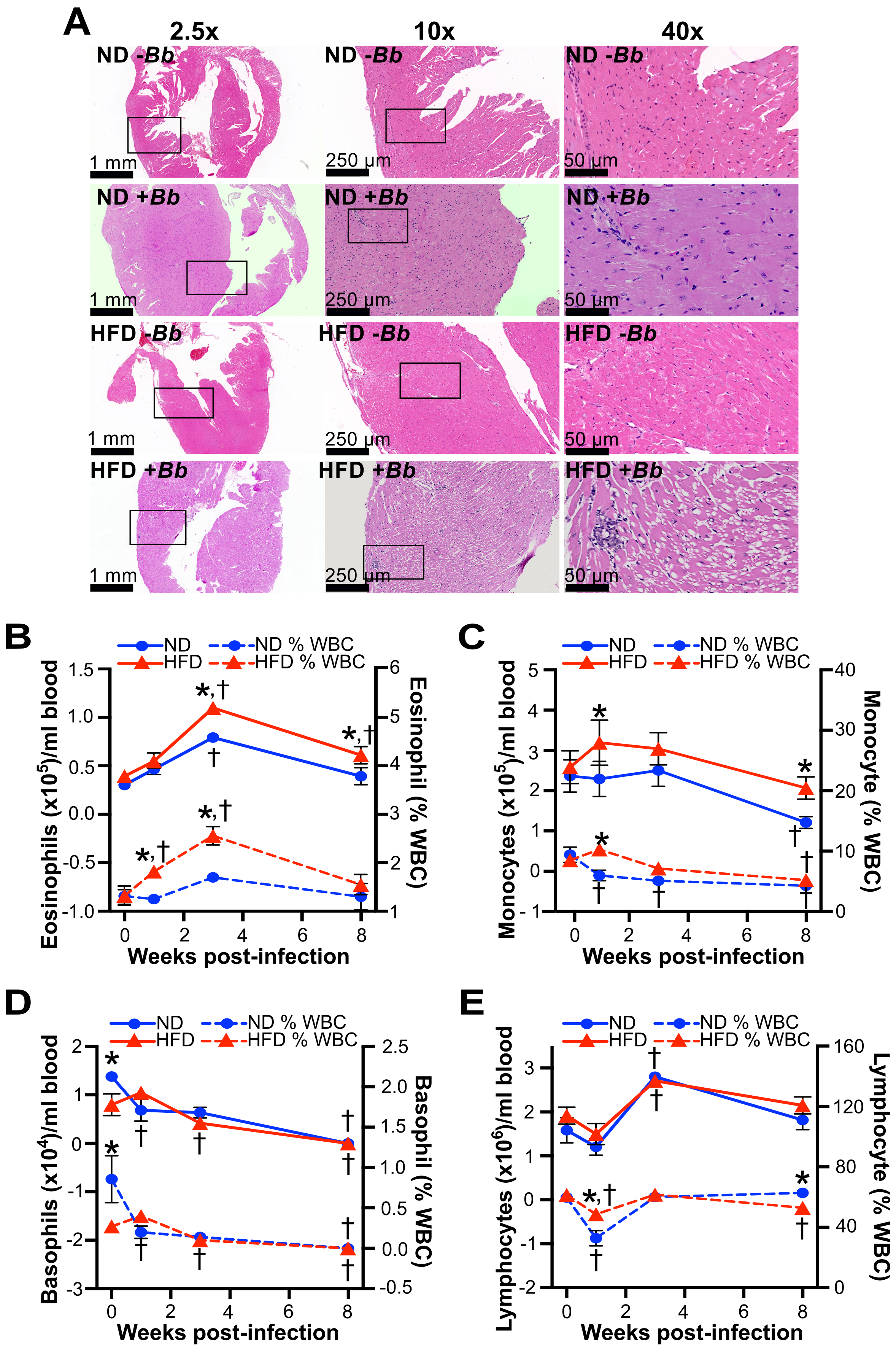


**FIG S3. Carditis and complete blood cell counts.** **(A)** Representative hematoxylin and eosin-stained sagittal heart sections used for carditis scoring by nuclei counting. Panel depicts male HeN mice infected with *Bb* strain N40. (**B-E**) Mean ±SEM cell count per ml of blood for eosinophils **(B)** monocytes **(C)** basophils **(D)** and lymphocytes **(E)** for experiments shown in **Fig. 3**. The percentage of each cell type in total WBCs is plotted as dashed lines and the percentage plotted on the right y-axis. Statistics: two-way ANOVA, Holm-Sidak post-test. p<0.05 ND vs HFD (*), infected vs uninfected (0 weeks) within the same diet group (†)


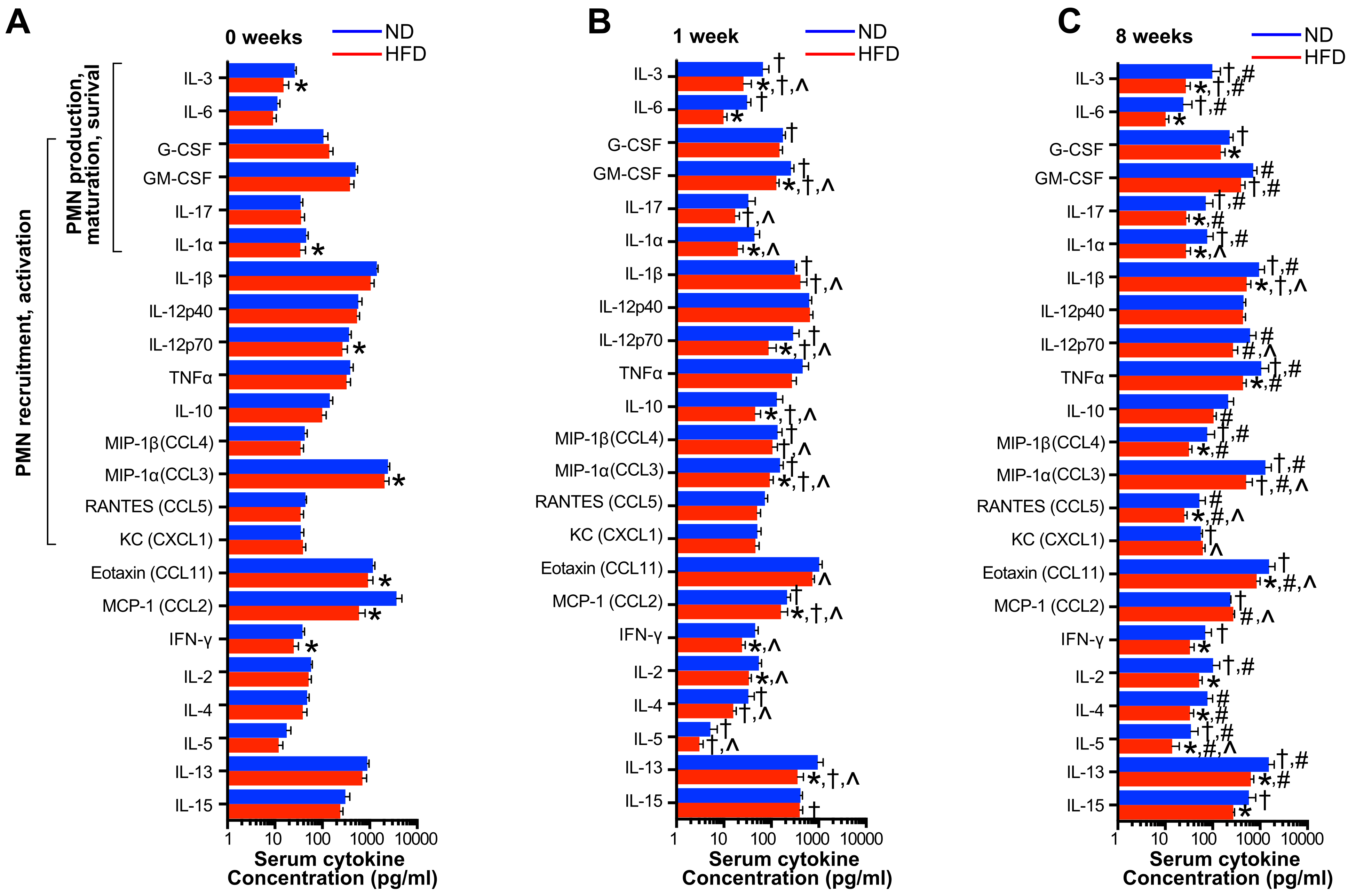


**FIG S4. Serum concentrations of individual cytokines in uninfected and *B. burgdorferi*-infected mice at 0, 1 and 8 weeks of infection.** Mean ±SEM serum cytokine concentrations for HeN male mice preconditioned on ND or HFD, infected with 1x10^4^ GCB726 and sacrificed at 0 **(A)**, 1 **(B)** and 8 **(C)** weeks post-infection. Cytokines involved in neutrophil production, maturation, survival, recruitment and activation are indicated. n=10-11 mice per diet group and time point. Statistics: 2way ANOVA of log-transformed values, with Holm-Sidak post- test. p<0.05 vs ND 0 week (^), 8 week vs 1 week within diet group (#), infected (1 or 8 weeks) vs uninfected baseline within diet group (†), HFD vs ND (*). Cytokine abbreviations: IL (interleukin), G-CSF (granulocyte colony-stimulating factor), GM-CSF (granulocyte macrophage colony-stimulating factor), IFN-γ (interferon γ), KC (keratinocyte chemoattractant), MCP-1 (monocyte chemoattractant protein 1), MIP-1α (macrophage inflammatory protein 1α), MIP-1β (macrophage inflammatory protein 1β), RANTES (Regulated on activation, normal T cell expressed and secreted), TNF-α (tumor necrosis factor α).


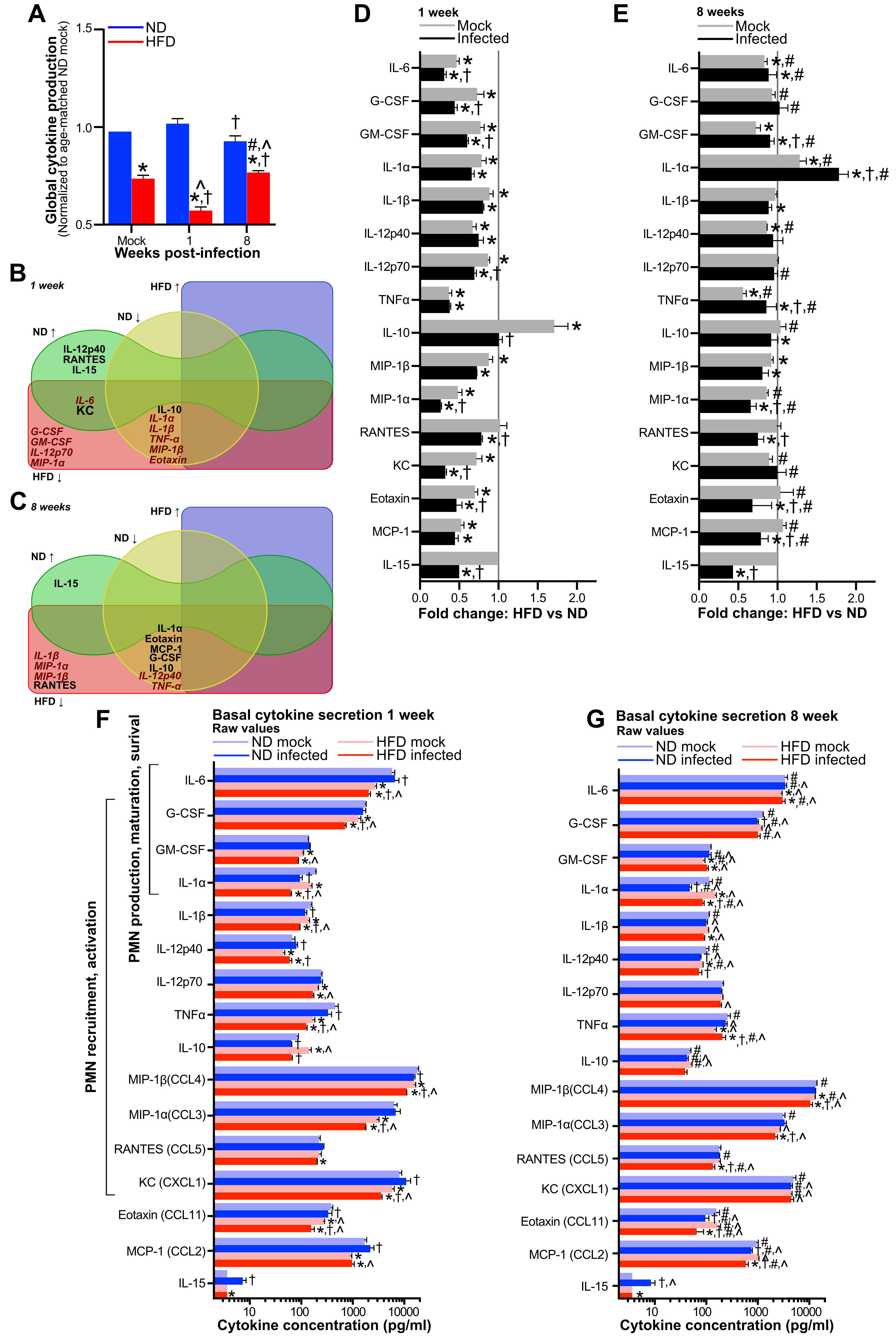


**FIG S5. Basal cytokine production by peritoneally recruited macrophages.** Basal cytokine production by peritoneal macrophages harvested from male HeN mice inoculated with bacterial cultivation medium alone (Mock) or 1 x 10^4^ GCB726 (Infected). **(A)** Mean ±SEM global basal cytokine production, normalized to age-matched ND mock controls. (**B-C**) Venn diagrams illustrating significantly (p<0.05) elevated (↑) or reduced (↓) production of cytokines at **(B)** 1 and **(C)** 8 weeks post-infection, compared to age-matched ND mock-infected controls. (**D-E**) Fold-difference in cytokine production for mock and infected HFD groups compared to mock and infected ND groups at **(D)** 1 and **(E)** 8 weeks post-inoculation. **(F-G)** Cytokine concentration in culture supernatant of unstimulated macrophages at 1 **(F)** and 8 **(G)** weeks post-infection of mice. For all panels, N>12 mice per experimental group and time point. Statistics: 2-way ANOVA of log-transformed data with Holm-Sidak post-tests. p<0.05: HFD vs ND within time point (*), vs age- and diet-matched mock control (†), vs ND mock 1 week (^), vs 1 week within the same diet (#).


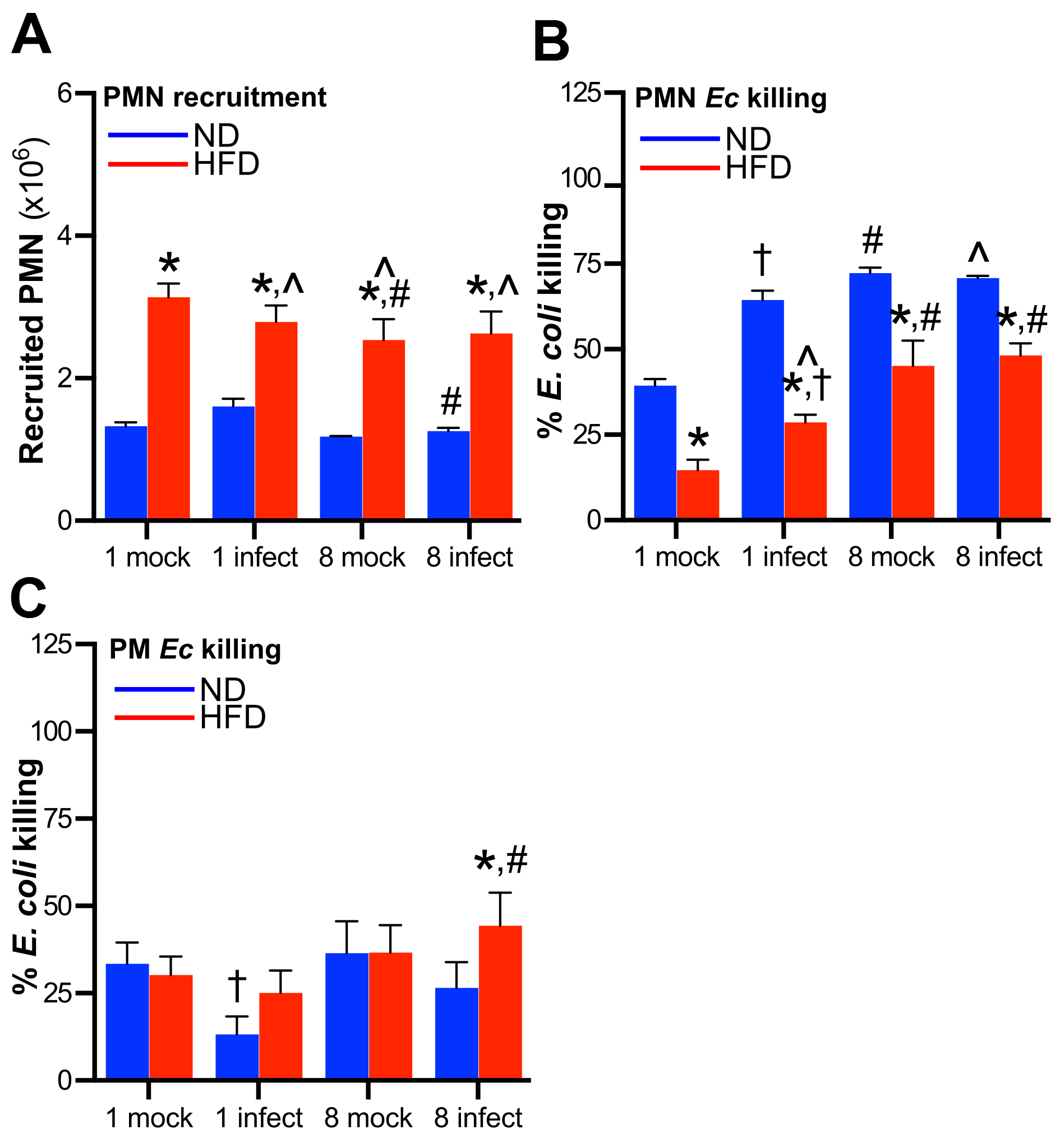
**FIG S6. Peritoneal neutrophil recruitment and *E. coli* killing by peritoneally-recruited neutrophils and macrophages.** Neutrophil (PMN) recruitment to peritoneum in response to sodium periodate **(A)** and killing of *E. coli* by peritoneally-recruited neutrophils (PMN: **B**) and by peritoneally-recruited macrophages (PM: **C**). All panels: Male HeN mice were inoculated with cultivation medium alone (Mock) or 1x10^4^ GCB726 (Infected) for 1 or 8 weeks before harvest of cells. N= 5 and 12-13 mice/ experimental group and time point for PMN and PM experiments, respectively. Summary values for all panels mean ±SEM. Statistics: 2-way ANOVA, Holm-Sidak post-test. p<0.05: HFD vs age-matched ND within infection group (*), vs age-matched Mock within diet group (†), vs age-matched ND mock (^), vs 1 week within diet and infection group (#).

**
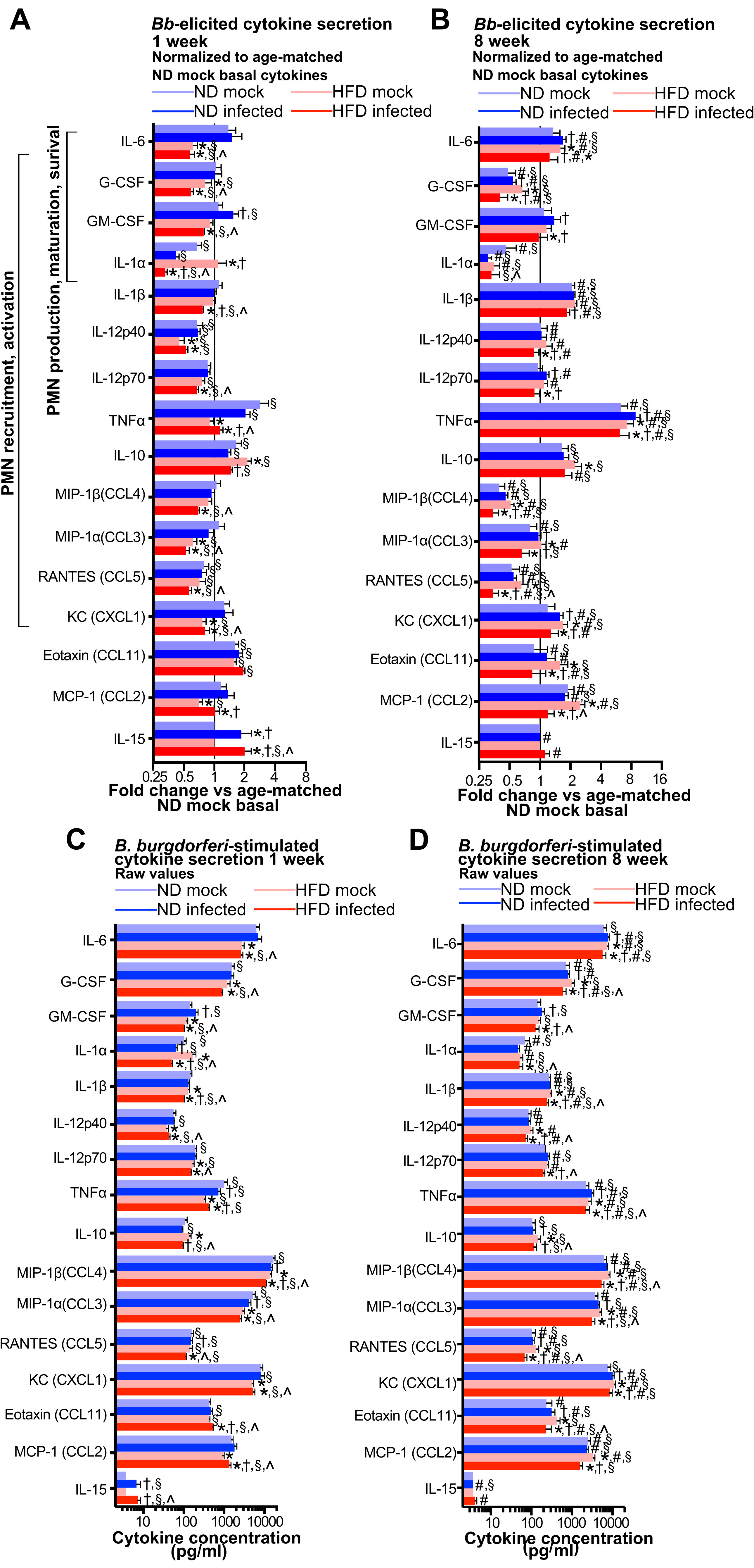
FIG S7. Normalized and raw levels of individual cytokines secreted by peritoneal macrophages in response to *B. burgdorferi* coincubation.** Mean ±SEM cytokine production by peritoneally-recruited macrophages harvested from age-matched male HeN mice inoculated with bacterial cultivation medium alone (Mock) or 1x10^4^ GCB726 (Infected) at 1 **(A, C)** and 8 **(B, D)** weeks post-inoculation. Values are normalized **(A-B)** to mean values for basal cytokine production in age-matched ND mock-infected groups to facilitate comparisons. Raw, non-normalized data are presented in C-D. N>12 mice/experimental group and time point. Statistics: 2way ANOVA of log-transformed data, Holm-Sidak post-tests. p<0.05 vs age-matched ND mock group (^), 8 week vs 1 week within diet and infection group (#), infected (1 or 8 weeks) vs uninfected within diet group (†), HFD vs ND (*), *B. burgdorferi*-coincubated vs ND mock basal (§).
